# Development and evaluation of a deep learning approach for modeling seasonality and trends in hand-foot-mouth disease incidence in mainland China

**DOI:** 10.1038/s41598-019-44469-9

**Published:** 2019-05-29

**Authors:** Yongbin Wang, Chunjie Xu, Shengkui Zhang, Li Yang, Zhende Wang, Ying Zhu, Juxiang Yuan

**Affiliations:** 1 Department of Epidemiology and Health Statistics, School of Public Health, North China University of Science and Technology, Tangshan, Hebei Province P.R. China; 2 Department of Occupational and Environmental Health, School of Public Health, Capital Medical University, Beijing, 100069 P.R. China

**Keywords:** Viral infection, Applied mathematics, Statistics

## Abstract

The high incidence, seasonal pattern and frequent outbreaks of hand, foot, and mouth disease (HFMD) represent a threat for millions of children in mainland China. And advanced response is being used to address this. Here, we aimed to model time series with a long short-term memory (LSTM) based on the HFMD notified data from June 2008 to June 2018 and the ultimate performance was compared with the autoregressive integrated moving average (ARIMA) and nonlinear auto-regressive neural network (NAR). The results indicated that the identified best-fitting LSTM with the better superiority, be it in modeling dataset or two robustness tests dataset, than the best-conducting NAR and seasonal ARIMA (SARIMA) methods in forecasting performances, including the minimum indices of root mean square error, mean absolute error and mean absolute percentage error. The epidemic trends of HFMD remained stable during the study period, but the reported cases were even at significantly high levels with a notable high-risk seasonality in summer, and the incident cases projected by the LSTM would still be fairly high with a slightly upward trend in the future. In this regard, the LSTM approach should be highlighted in forecasting the epidemics of HFMD, and therefore assisting decision makers in making efficient decisions derived from the early detection of the disease incidents.

## Introduction

Hand, foot and mouth disease (HFDM) is a common acute infectious disease in children, the majority (91%) of whom are under 5 years^1^. Most show mild symptoms mainly characterized by fever and rash on the hands, feet and mouth. A small minority have more severe complications, such as myocarditis, pulmonary edema and aseptic meningoencephalitis, some of which are fatal^2^. The infections are predominantly caused by coxsackievirus A16 (CVA16) and human enterovirus 71 (EV71), although other viruses can be involved^3^. The viruses are thought to be predominantly transmitted from child to child by direct and indirect contact, including droplets, droplet nuclei, dust, water and food^1,4^. Furthermore, approximately half of the individuals may be coinfected with more than one pathogenic agent^5^. HFMD was first reported in New Zealand in 1957, and since then, millions of cases and many outbreaks have been reported worldwide^6^. However, the worldwide epidemiology of HFMD has dramatically changed during the past decade, especially in East and Southeast Asian countries such as China, Brunei, Malaysia, Mongolia, Singapore and Vietnam^7^, where epidemics and numerous large-scale outbreaks of HFMD have occurred, resulting in enormous burdens of disease and global public health concerns^3,8,9^.

In mainland China, after the first reported case of HFMD in Shanghai in 1981^10^, several outbreaks have been reported and have caused the deaths of numerous children^3^. Moreover, HFMD affects more than two million children annually in mainland China^11^, and the number of cases and deaths caused by HFMD sporadics, epidemics and outbreaks invariably tops the list of monitored class C diseases every year since HFMD was designated as a notifiable disease in 2008^3,12^. Since 2016, although an available vaccine that only plays a role in the infection caused by the EV71 virus has been introduced to prevent HFMD^13^, the potentially worsening trend in the reported cases of HFMD has not been reversed. Importantly, it is estimated that there is an increasing risk of ongoing HFMD recurrence in China in recent years^14^ and HFMD still continues to exert a significant influence on the general susceptible population. Early detection and advanced warning for the timing, extent and duration of HFMD epidemics will be particularly valuable in formulating effective prevention and intervention strategies to minimize the damage caused by the infection^15^. Therefore, a reliable forecasting technique to track the temporal patterns of HFMD is needed.

The existing early warning models for forecasting the morbidity and mortality of infectious diseases mainly consist of linear and nonlinear models, along with their hybrids^16^. Additionally, the autoregressive integrated moving average (ARIMA) model is one of the best linear models in terms of performance for a specified time series^17^; the nonlinear autoregressive neural network (NAR) approach is among the nonlinear models with arbitrarily expected accuracy that can effectively extract the meaningful dynamic information of a data sequence^16^. Both the ARIMA and NAR models are well suited to study the future trends of the morbidity or mortality time series of diseases with stationary short-term dependencies based on the aggregated long trajectories^16^. However, long-term trajectory modeling, which is most often encountered in epidemiological prediction, is frequently characterized by non-stationary long-term lags over time. Additionally, when employing an NAR model to connect the preceding information located into the time-varying series to the present assignment, with the growing gap between the past inputs and estimated outputs, the NAR technique will encounter a vanishing or exploding gradient problem during training, which makes it difficult to develop the long-term dependence structure in a time series^18^. The long short-term memory (LSTM) architecture, a type of deep learning network that has been extensively studied and applied to quite a few frontier fields, such as voice recognition^19^, video classification^20^ and speech synthesis systems^21^, comprises a cluster of recurrently connected subnets that allow the LSTM method to store and access information over long periods of time, hence mitigating the vanishing or exploding gradient problem^22^. However, there is a current lack of research focusing specifically on the applicability of LSTM model in the forecasting of infectious diseases with time series analysis. Therefore, motivated by the merits of the LSTM model, the burden of HFMD and the persistently high incidence in mainland China, we aim to forecast the notified incident cases of HFMD with a LSTM model. Meanwhile, the simulating and predictive abilities of the LSTM model were compared with two especially useful estimation models, including the ARIMA and NAR methods, to seek the best-fitting time series modeling technique for HFMD, which will be of great help in initiating guidance planning and effective intervention measures for HFMD-prevention in mainland China.

## Results

### General information

**Figure 1:**
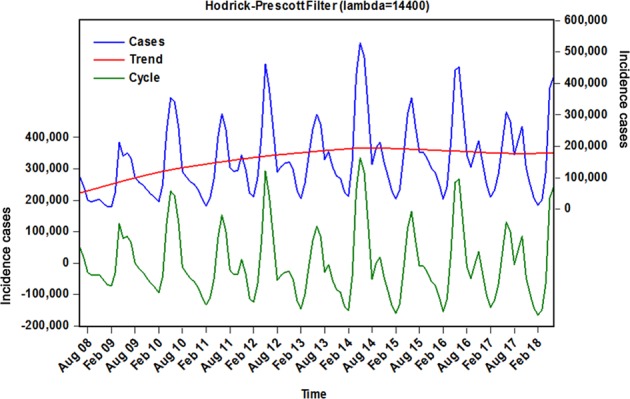
Time series of monthly HFMD observed series and decomposition using Hodrick-Prescott filter in mainland China from June 2008 to June 2018.

The time series of notified cases of HFMD included 121 observations from June 2008 to June 2018, and a total of 19,218,824 cases were reported. The monthly average of case notifications was 158,834, which led to an annualized average morbidity rate of 133,894 cases per 100,000 population with a standard error of 10,825 cases during the whole period. Between June 2008 and June 2017, 17,072,500 cases occurred, and the number of morbidity cases increased from 102,223 to 308,789, with an overall increase of 202.074% throughout the past decade. The highest incidence peak of 528,777 cases was observed in May 2014, which was a marginal increase of 417.278% compared to June2008. When the Hodrick-Prescott (HP) decomposition approach was performed to smooth the short-term monthly effect of the observed incidence case series from June 2008 to June 2018, it was found that there were clear seasonal peaks, specifically in May and June of every year, a trough in January and February and an evident 12-month cyclical process. In addition, notwithstanding a slight decline that existed between January 2014 and June 2018, there were still a substantially large number of reported cases each year (Fig. 1 and Supplementary Fig. S1).

### The best-performing SARIMA model

**Figure 2:**
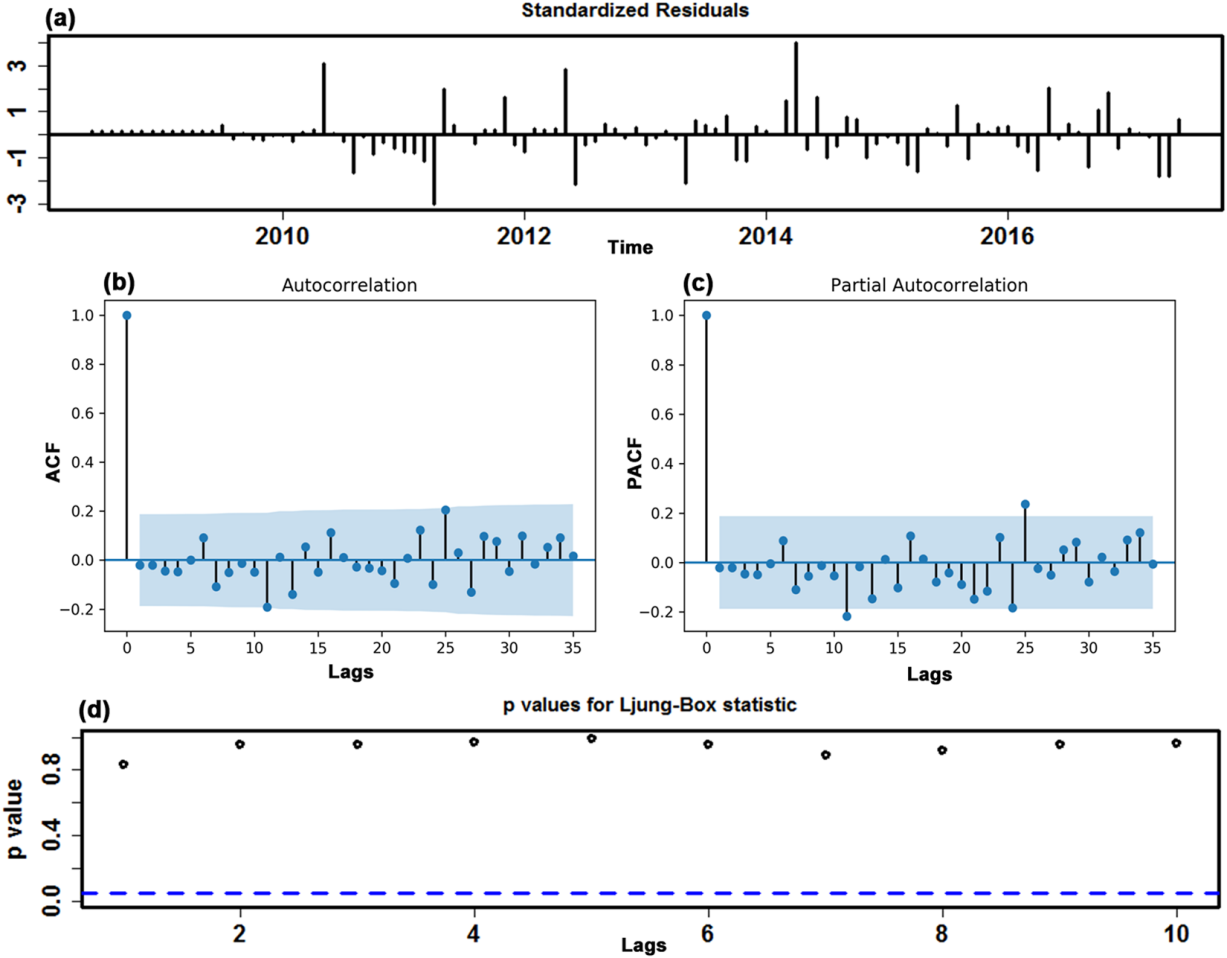
The resulting plots of fit goodness tests from SARIMA(1,1,2)(1,1,0)_12_ model for HFMD notified cases series. (**a**) Standardized residuals. (**b**) Autocorrelation function (ACF) graph of errors across varying lag times. None of the autocorrelation coefficients are beyond the 95% confidence intervals in this residual series. (**c**) Partial autocorrelation function (PACF) graph of errors. (**d**) Q-statistic *P*-values. There are large *P* values at the significance level of 5%. Diagnostic checking indicates the chosen SARIMA specification can provide a reasonable approximation to the HFMD notified cases series.

Before modeling, the original in-sample observations were examined using the augmented Dickey-Fuller (ADF) test (ADF = −2.3318, *P* = 0.1642), which is indicative of an obviously non-stationary morbidity case series. Therefore, according to the results from the HP filter and ADF test, the first-order seasonal and non-seasonal differences were used to stabilize the variance and mean to suit the modeling requirement of a stationary sequence (ADF = −4.4129, *P* = 0.0006). Analysis of the spikes in the ACF and PACF plots at varying lags with the transformed incidence case series for HFMD resulted in the selection of several candidate models by trial and error to further detect the best-performing specification (Supplementary Figs S2 and S3). Finally, taking the error correlations between the ACF and PACF plots comprehensively and taking the AIC, AICc and SBC into consideration (Fig. 2 and Supplementary Table S1), the preferred model of SARIMA(1,1,2)(1,1,0)_12_ was generated with minimized AIC, AICc and SBC values of 2339.41, 2340.08 and 2352.23, respectively. When the error correlations at lags fell into the estimated 95% confidence bounds (Fig. 2b,c), the Ljung-Box *Q* test showed that the residuals from the SARIMA model obtained desirable white noise (Fig. 2d and Table 1), and the LM test suggested that no ARCH effect was found at various lags in the residual series (Table 2). Furthermore, the test results of the estimated parameters were also all significant (Supplementary Table S1). Nevertheless, the only complication was that the Q-Q plot of the residuals showed a clear departure from normality at the tails (Supplementary Fig. S4). The specified equation of the SARIMA model was expressed as (1-B)(1-B^12^)X_t_ = (1 + 0.524B + 0.394B^2^)ɛ_t_/(1 − 0.434B)(1 + 0.6B^12^). In the same way, the reported case series of HFMD from June 2008 to December 2016 and December 2017 was utilized to account for the robustness of the model. The best-fitting SARIMA method constructed using the first 103 in-sample observations was identified as a SARIMA(1,0,1)(1,1,1)_12_ form, and Supplementary Figs S5–S8 and Tables S2–S5 provide the results of the diagnostic tests for this optimal SARIMA approach; the best-simulating SARIMA approach obtained with the first 115 observed points was still identified as a SARIMA(1,0,1)(1,1,1)_12_ form, and the identified parameters and diagnostic tests for the preferred approach are given in Supplementary Figs S9–S12 and Tables S6–S9. Then, these optimal methods chosen were utilized to calculate out-of-sample predictions.

**Table 1:** Ljung-Box *Q* test of the residuals for the selected three optimal models at different lags.

Lags	SARIMA model		NAR model		LSTM model	
	Box-Ljung *Q*	*P*	Box-Ljung *Q*	*P*	Box-Ljung *Q*	*P*
1	0.046	0.830	0.963	0.326	1.324	0.250
3	0.319	0.956	3.489	0.322	2.818	0.421
6	1.578	0.954	5.051	0.537	3.143	0.791
9	3.295	0.952	6.941	0.643	3.441	0.944
12	8.170	0.772	7.442	0.827	7.141	0.848
15	11.330	0.729	11.443	0.721	12.800	0.618
18	13.122	0.784	11.626	0.866	15.252	0.645
21	14.728	0.836	14.941	0.826	15.355	0.805
24	18.265	0.790	16.648	0.863	16.889	0.853
27	26.991	0.464	17.448	0.920	19.358	0.857
30	29.634	0.485	19.393	0.931	21.247	0.880
33	31.664	0.534	21.815	0.932	23.716	0.883
36	50.369	0.056	24.575	0.925	27.236	0.853

**Table 2:** ARCH effect of the observations and residuals of the selected three models with LM test at various lags.

Lags	Observed values		SARIMA model		NAR model		LSTM model	
	LM-test	*P*	LM-test	*P*	LM-test	*P*	LM-test	*P*
1	54.599	<0.001	1.869	0.172	0.262	0.609	0.002	0.961
3	73.920	<0.001	2.669	0.446	2.823	0.420	0.440	0.932
6	72.753	<0.001	3.575	0.734	2.972	0.812	3.338	0.765
9	71.185	<0.001	4.703	0.859	14.741	0.098	5.216	0.815
12	73.136	<0.001	14.080	0.296	11.422	0.493	7.294	0.838
15	71.275	<0.001	14.566	0.483	30.219	0.011	10.241	0.804
18	69.282	<0.001	15.566	0.623	32.252	0.020	13.735	0.746
21	67.505	<0.001	20.645	0.481	34.845	0.029	0.7462	0.881
24	73.785	<0.001	21.094	0.633	34.774	0.072	14.815	0.926
27	71.487	<0.001	25.263	0.560	36.360	0.108	18.172	0.898
30	68.830	<0.001	27.984	0.571	38.706	0.133	19.899	0.919
33	66.153	<0.001	32.378	0.498	41.990	0.136	22.538	0.915
36	64.210	<0.001	33.766	0.575	46.280	0.117	23.631	0.944

### The best-performing NAR model

**Figure 3:**
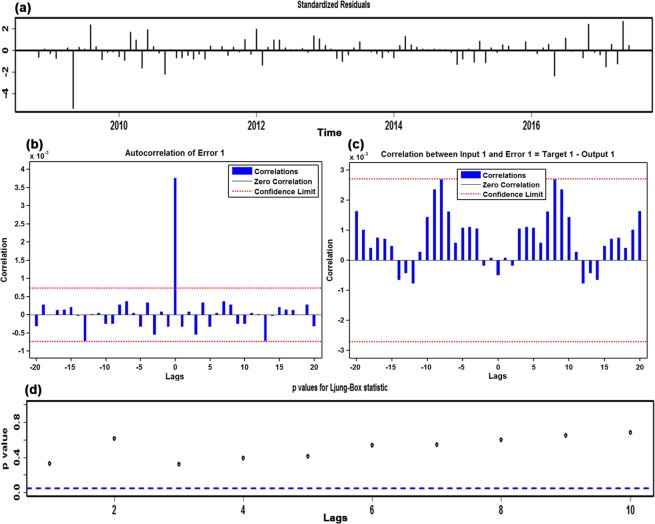
The resulting plots of fit goodness tests from the best-fitting NAR model for HFMD notified cases series. (**a**) Standardized residuals. (**b**) Autocorrelation function (ACF) plot of errors across varying lag times. All of the autocorrelations fail to be beyond the estimated 95% uncertainty bounds around zero across varying lag times apart from the one from ACF plot at zero lag that should occur. This manifests that the network appears to have captured the dependence hidden behind the HFMD notified cases series. (**c**) Input-to-error correlation plot for varying lags. The input-error cross-correlation function illustrates how the residuals are interrelated with the series of x(t). All of the correlations fall within the confidence bounds around zero, which hints the developed model is a perfect specification. (**d**) Q-statistic *P*-values. Analyses form the plots demonstrate the constructed model is adequate in excavating the information of this time series.

**Figure 4:**
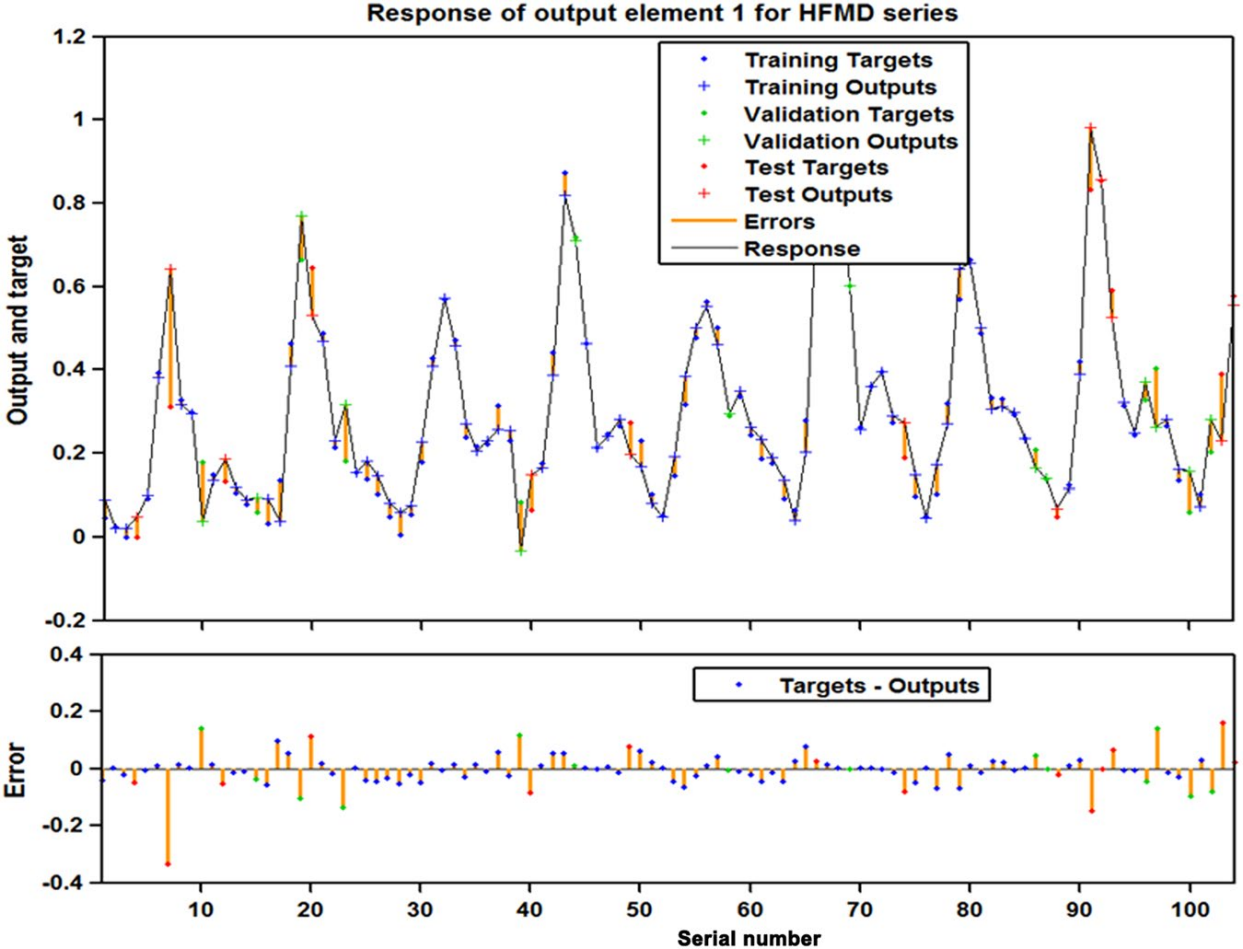
The response of output and target for HFMD time series at various time points. This plot exhibits which time points are elected as the training, validation and testing subsets, along with their corresponding errors between inputs and targets. In view of the small errors, a further suggestion that the fitting is fairly accurate.

To obtain an optimum NAR model, the hidden units and feedback delays, ranging from 10 to 35 and from 2 to 8, respectively, were iteratively examined within in-sample data points. Ultimately, we identified the best-fitting model with 18 hidden neurons and 5 feedback delays dependent on the comprehensive optimum performance indices aside from the fact that a fat-tailed distribution, compared to the normal distribution, should be utilized (Supplementary Fig. S13). As presented in Supplementary Fig. S14, in the preferred model, the minimum MSE values for the training, validation and testing datasets and for the entire dataset were 0.0011, 0.0074, 0.0144 and 0.0038, respectively; the maximum R values of the training, validation, testing subsets and entire dataset were 0.987, 0.931, 0.920 and 0.963, respectively. Moreover, the input-to-error correlations and autocorrelations of the produced residuals were never beyond the estimated 95% uncertainty limits around zero across varying lag times, apart from the one in the ACF plot at lag zero that should occur (Fig. 3 and Table 1). The LM test suggested that the ARCH effect that existed in the original data largely minimized the errors of the NAR model (Table 2). In addition, the response plot of the estimated values from the randomly selected training, validation and testing datasets against their corresponding original observations at different time points showed that the optimal approach could simulate the data points included in the three grouped subsets well because of the small residuals that were mostly located between −0.2 and 0.2 (Fig. 4). Similarly, according to the modeling steps described above, in these two robustness-test datasets, the best-presenting technique fit to the dataset between June 2008 and December 2016 was such an NAR model with 17 hidden neurons and 5 feedback delays. The identified layer architecture and statistical measures for this preferred network are displayed in Supplementary Figs S15–S19 and Tables S4, S5 and S10. The best-fitting model developed utilizing the data from June 2008 to December 2017 was an NAR model with 19 hidden neurons and 6 feedback delays, and with regard to the optimal network, all further diagnostic results can be seen in Supplementary Figs S20–S24 and Tables S8–S10. Afterwards, these best-performing networks were employed to conduct out-of-sample forecasting, and the simulated and forecasted values obtained were back-transformed to the original scale because they were computed on the transformed scale.

### The best-performing LSTM model

**Figure 5:**
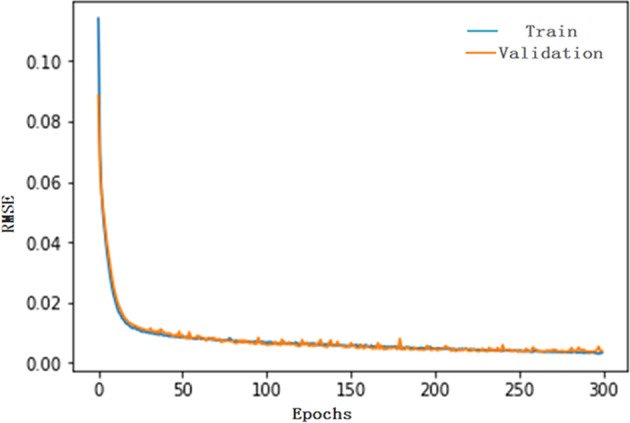
The training and validation performances for LSTM model at 300 epochs. This plot documents that no overfitting is observed during the training process due to the similar downward trend before 300-step iterations.

**Figure 6:**
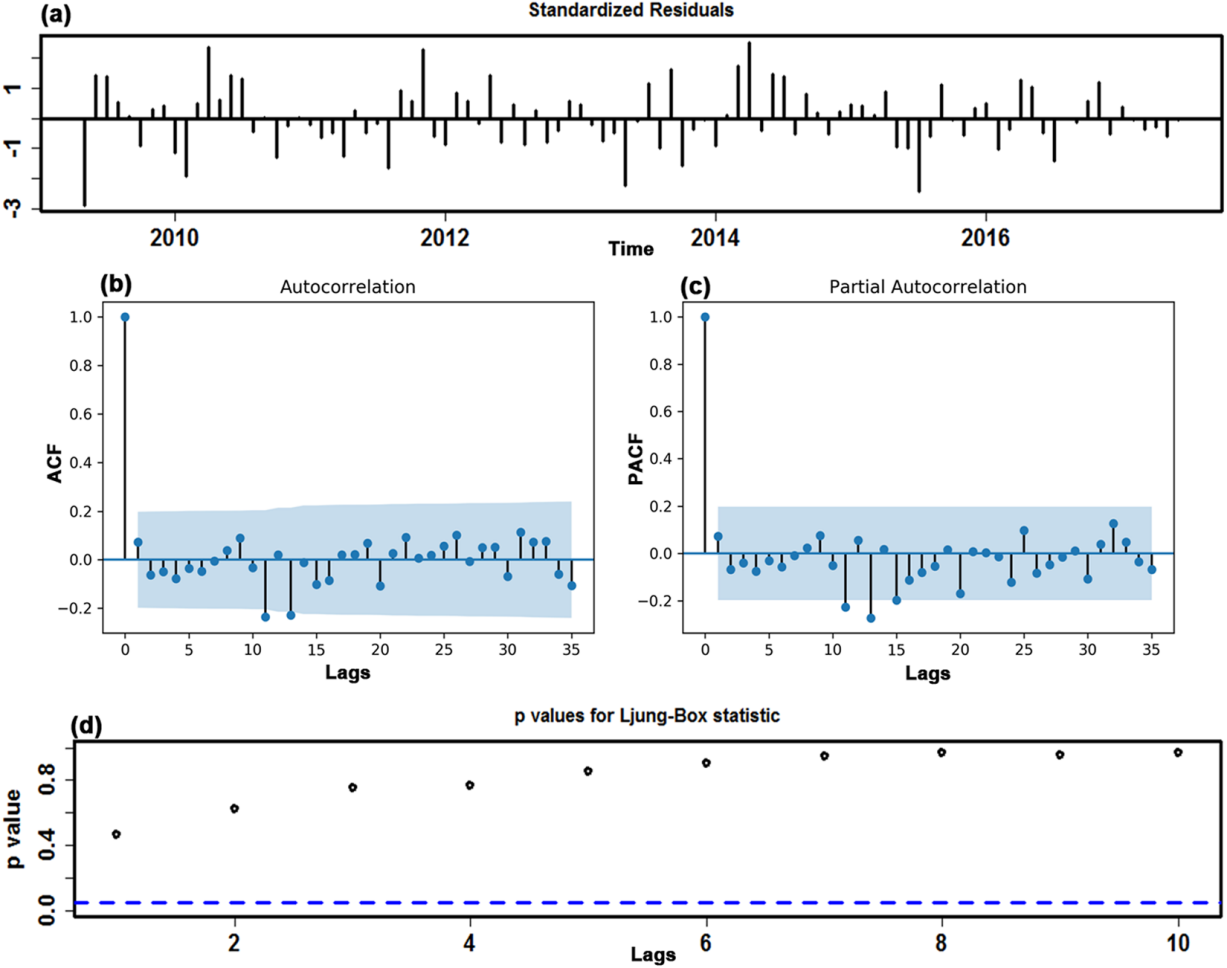
The resulting plots of fit goodness tests from the LSTM model for HFMD notified cases series. (**a**) Standardized residuals. (**b**) Autocorrelation function (ACF) plot of errors across varying lag times. The ACF plot of forecasted errors reveals no individually evident autocorrelation at varying lags except for the two points occurring at lags 11 and 13. For these two lagged points out of the estimated 95% confidence limit, they are also reasonable as this phenomenon can easily happen by chance alone. (**c**) Partial autocorrelation function (PACF) plot of residuals. (**d**) Q-statistic *P*-values. As shown, All *P*-values are larger than 0.05. These diagnostics manifest that the network is well suited to the dataset.

Generally, the LSTM network with 1 hidden layer surrounded by 1 to 7 hidden units can satisfy the need for time series modeling. Consequently, to attain the optimal modeling parameters for the HFMD series, the range of time steps was set to 1 to 20, and the LSTM model was conducted repeatedly utilizing the activation sigmoid function with time steps ranging from 1 to 20 together with a batch size of 1 using the Adam optimizer technique to stabilize the argument updates to help minimize the loss function of the RMSE. All candidate models were iterated through 300 epochs. Ultimately, we identified that the best-simulating model with 1 hidden layer containing 5 hidden neurons and 11 time steps relied on a minimum training score of RMSE = 0.0031 and a testing score of RMSE = 0.0038 and with maximum R values of the training and validation subsets and of the overall data (0.972, 0.982 and 0.974, respectively) (Supplementary Fig. S25). In addition, as presented in Fig. 5, no overfitting occurred during the training process because of the similar downward trend between the testing and validation datasets before 300-step iterations. The ACF plot of the produced errors revealed no individually evident autocorrelation at varying lags except for the one at lag zero (Fig. 6b), as shown in the normal Q-Q plot (Supplementary Fig. S26), indicating that the forecasted residuals of the LSTM model were normally distributed to a great extent. The Ljung-Box *Q* test showed that errors did not depart from the assumptions of stochastic white noise (Fig. 6d and Table 1), and the LM test demonstrated that the volatility that existed in the actual observations was essentially eliminated in the residuals of the selected LSTM model as well (Table 2). Therefore, the model chosen is adequate in capturing the dynamic dependences of this time series. Likewise, the in-sample observations used to test the robustness of the model were applied to determine the optimal LSTM network as noted earlier: the network with 1 hidden layer including 6 hidden neurons and 12 time steps was constructed based on the data series from June 2008 to December 2016 was the best-performing; the results of the diagnostic tests for this network are summarized in Supplementary Figs S27–S30 and Tables S4, S5 and S11. The network with 1 hidden layer containing 5 hidden neurons and 11 time steps built on the basis of the data series from June 2008 to December 2017 should be regarded as the best-performing. Supplementary Figs S31–S34 and Tables S8, S9 and S11 offer systematic diagnostic tests for this preferred network. After the optimal LSTM approaches are selected, they can be employed to predict the epidemic trends of HFMD in the upcoming years.

### Comparative analysis

**Figure 7:**
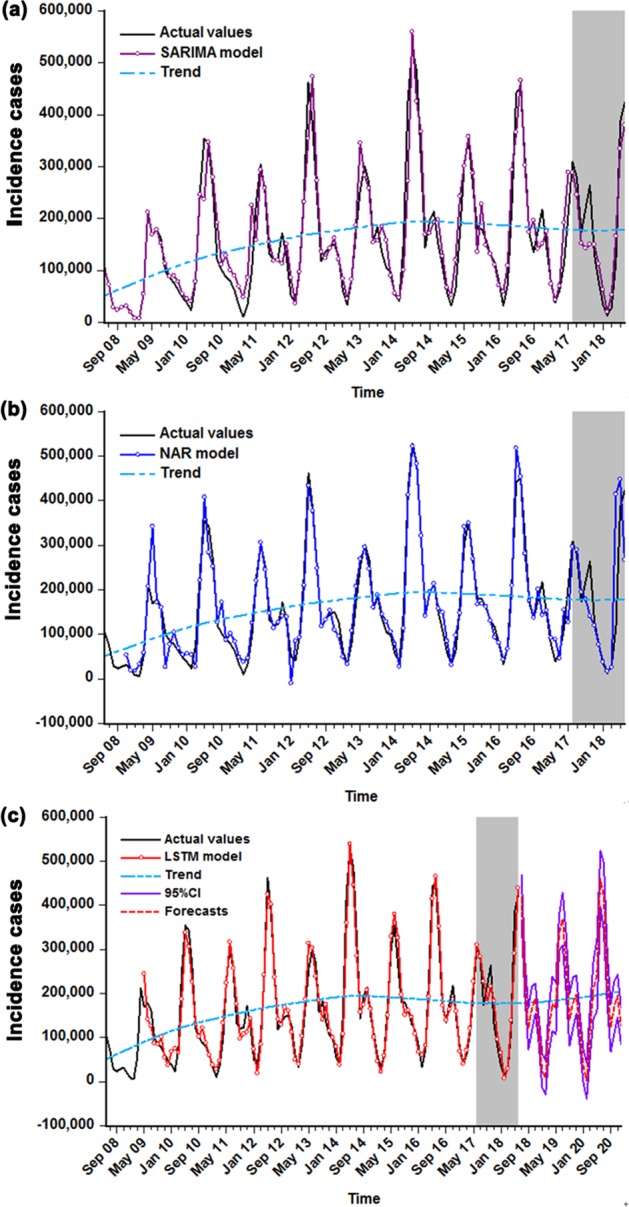
The comparisons of in-sample simulations and out-of-sample predictions among these three models selected. (**a**) Comparison between the actual observations and the results from the SARIMA model. (**b**) Comparison between the actual observations and the results from the NAR model. (**c**) Comparison between the actual observations and the results from the LSTM model. The shaded area represents the validation sets from July 2017 to June 2018, in which the comparative results between the values predicted by the three selected models and the actual suggest that the curve forecasted by LSTM model is more proximal to the actual. As presented in (**c**), the red dotted line stands for the trends from July 2018 to December 2020 projected by the LSTM method.

Multiple statistical measures were applied to compare the in-sample simulation and out-of-sample predictive accuracies among the three methods. Compared to the SARIMA and NAR models, the minimum values of measures concerning the facets of training and testing were observed in the LSTM technique, aside from the MAE in the simulated stage of the NAR model, and were fitted to the robustness-test dataset from June 2008 to December 2017 (Table 3). For these three constructed models, overall, the curves simulated and predicted by the LSTM model were closer in proximity to the actual values as well (Fig. 7), which further implies that the epidemic trajectories of HFMD can be captured reasonably well by the LSTM technique. Hence, the LSTM technique was re-modeled to recursively achieve multistep-ahead predictions from July 2018 to December 2020 (Table 4); it appears that a slightly potential rising risk in the incident cases of HFMD will be observed in the forecasting period. In the meantime, the uncertainty bands for the resultant forecasts were estimated utilizing simulation of 100 future possible paths by performing bootstrapping with the number of samples of 1000^23,24^ (Table 4 and Supplementary Table S12).

**Table 3:** The comparison results of in-sample simulating and out-of-sample predicted performances for the three models.

Models	Simulated performance			Predicted performance		
	MAE	MAPE	RMSE	MAE	MAPE	RMSE
**In-sample observations from June 2008 to December 2017**				**6 out-of-sample predictions**		
SARIMA	30610.689	0.223	44383.404	40101.500	0.387	51150.446
NAR	19652.784	0.222	29740.835	21004.106	0.361	27364.566
LSTM	21548.164	0.203	27716.239	19505.800	0.221	25820.167
**Reduced percentages (%)**						
LSTM vs. SARIMA	29.606	8.969	37.553	51.359	42.894	49.521
LSTM vs. NAR	−6.192	8.520	4.562	3.736	36.176	3.019
**In-sample observations from June 2008 to June 2017**				**12 out-of-sample predictions**		
SARIMA	30268.335	0.275	43330.873	41542.355	0.366	49978.944
NAR	20678.368	0.272	31998.251	61130.494	0.373	105803.807
LSTM	20584.538	0.192	26364.913	32451.557	0.262	41678.916
**Reduced percentages (%)**						
LSTM vs. SARIMA	31.993	30.182	39.154	21.883	28.415	16.607
LSTM vs. NAR	0.310	29.091	13.001	69.035	30.328	128.304
**In-sample observations from June 2008 to December 2016**				**18 out-of-sample predictions**		
SARIMA	30136.495	0.211	43456.811	43434.889	0.479	53852.232
NAR	19957.815	0.186	29345.366	73291.494	0.629	88387.215
LSTM	19931.051	0.176	25155.016	39729.413	0.351	50951.064
**Reduced percentages (%)**						
LSTM vs. SARIMA	33.864	16.588	42.115	8.531	26.722	5.387
LSTM vs. NAR	0.089	4.739	9.643	77.270	58.038	69.516

**Table 4:** The predictive incident cases of HFMD using the best-presenting LSTM technique from July 2018 to December 2020.

Time	Predictions	95% uncertainty bands	Time	Predictions	95% uncertainty bands
Jul-2018	417719	[366119, 469320]	Oct-2019	179164	[120755, 237573]
Aug-2018	232278	[192745, 271810]	Nov-2019	197632	[153917, 241346]
Sep-2018	120464	[73475, 167453]	Dec-2019	116959	[80180, 153739]
Oct-2018	164881	[114495, 215267]	Jan-2020	36199	[−650, 73047]
Nov-2018	188281	[153866, 222697]	Feb-2020	2426	[−38061, 42912]
Dec-2018	122213	[92903, 151522]	Mar-2020	115231	[61218, 169245]
Jan-2019	25824	[−12713, 64359]	Apr-2020	210295	[144546, 276044]
Feb-2019	8856	[−29540, 47252]	May-2020	240488	[159436, 321540]
Mar-2019	118917	[66934, 170900]	Jun-2020	460614	[397661, 523566]
Apr-2019	176434	[114298, 238571]	Jul-2020	419429	[342643, 496216]
May-2019	165857	[107939, 223776]	Aug-2020	232450	[174459, 290441]
Jun-2019	339025	[299026, 379023]	Sep-2020	121814	[67407, 176222]
Jul-2019	369045	[309402, 428689]	Oct-2020	168242	[108510, 2279730]
Aug-2019	272607	[207759, 337456]	Nov-2020	194318	[145616, 243020]
Sep-2019	140126	[81893, 198359]	Dec-2020	126442	[85527, 167356]

## Discussion

Since 2008, HFMD has regularly captured wide attention owing to both its high incidence and potential health hazards for millions of children, along with substantial losses to economy every year in mainland China^8^. It is imperative that specific control and intervention planning be introduced and set by the related public health agencies to handle such a wide-ranging issue. However, vital to any initiation planning of HFMD is an accurate prediction of its future temporal patterns. Early detection of HFMD epidemics based on models such as SARIMA and NAR has been a profitable technology for facilitating prevention strategies more effectively^10,11,17^. Therefore, in view of the LSTM model’s flexible capacity to learn what to store and what to abandon during information-processing^25^, to the best of our knowledge, this attempt is the first using an LSTM approach to model the long trajectory behaviors of HFMD incidence in mainland China. Our results imply that the LSTM method has the potential to obtain a clearer perspective of epidemic trends than the SARIMA and NAR models built on the specified predictive horizons. Notably, the LSTM method can make the outcome measurements of MAE, MAPE and RMSE markedly drop by 31.993%, 30.182% and 39.154%, respectively, in the training dataset, and can make their counterparts in the testing dataset decrease by 21.883%, 28.415% and 16.607%, respectively, compared to the SARIMA model. In contrast to the NAR model, the decreased percentages in the training subset for the three indices listed above are 0.310%, 29.091% and 13.001%, respectively, and their counterparts in the testing subset are 69.035%, 30.328% and 128.304%, respectively. Likewise, according to Fig. 7, it was found that the upward or downward trend simulated and forecasted by the LSTM model provides a more reasonable approximation to the reported points, especially for the identification of high incidence peaks, indicating that the LSTM model can adequately capture the essence of the dependence hidden behind the notified case series of HFMD. Similarly, in the two datasets used to account for the uncertainty in the model, the accuracy measurement indices in the LSTM approach also display the lowest error rates among these chosen, optimal methods, particularly in the 18-step ahead predictions, and the performances are as good as can be expected. The scale-dependent measure of MAE showed a lower value in the simulated stage of the NAR method than in the LSTM technique in the 6-step predictions; this result mainly arises from the fact that there are several simulated values that are far away from the observed values. Moreover, it was confirmed that the NAR model is effective in capturing the short-term dynamics of the data. In short, in comparison with the SARIMA and NAR models, the established LSTM approach not only can better explain the seasonal and trend characteristics of HFMD but also is robust with respect to medium-term and long-term forecasts. Apparently, this network can act as an effective tool for recognizing the temporal levels of HFMD incident cases in mainland China in upcoming years. Similar to the recent literature, which has also found that the LSTM method provides good forecasting power for air pollutant concentrations^26^, financial time series^27^ and harmful algal blooms in rivers^28^. From this point of view, our LSTM technique appears to be worthy of being popularized for forecasting the incidence case series of HFMD in other settings in China and even a wide range of simulation applications, such as for all types of contagious diseases or in all time series analyses; however, this conclusion requires further verification. It should, however, be noted that with the increasing development of hybrid techniques, numerous combined methods incorporating linear approaches such as the SARIMA method^17^, the gray GM(1,1) model^29^, the error-trend-seasonal model^30^ and the exponential smoothing model^31^ and nonlinear techniques such as the back propagation neural network approach^32^, the generalized regression neural network method^33^ and the radical basis function technique^32^ have already been adopted to serve as early warning tools for infectious diseases, and most have obtained satisfactory results. Consequently, much work will be required to explore the preferred models for detecting and analyzing HFMD morbidity cases in mainland China. In addition, in terms of the modeling measures (MAE, MAPE and RMSE), we found that the simulating and forecasting efficacies of the NAR method were slightly superior to the SARIMA method in the short-term (6-step) predictions, which is consistent with the earlier studies performed by Zhou *et al*.^34,35^, yet is incongruous with the study involving modeling the prevalence of schistosomiasis in Qianjiang^16^; in the medium-term (12-step) and long-term (18-step) prediction stages, the NAR method underperforms the SARIMA model in the testing dataset, but this result is not in line with previous work predicting the morbidity of hemorrhagic fever with renal syndrome^36^ and the daily number of new admission inpatients^35^. It seems possible that these contrasting results are due to the following: the various characteristics of infectious diseases from different regions and the NAR approach suffer from overfitting, which is a defect inherent in the ANN methods. However, during MATLAB training, a default technique of early stopping was adopted to improve generalization and avoid overfitting. Therefore, being further suggestive of the necessity of constructing forecasting techniques for different infectious diseases in various settings and at different time periods, it is superior to the NAR technique in short-term forecasting.

It is well established that accurate identification of high-risk seasonality plays a pivotal role in timely implementation of prevention strategies and the reasonable allocation of resources for HFMD^6^. In our report, HFMD could occur throughout the year, and larger epidemics could be regularly found every 2 to 3 years. Similar trajectory behaviors were also reported in the studies involved in the regions of Vietnam^37^, Malaysia^38^, Hong Kong^39^, Taiwan^40^ and Singapore^41^, but the underlying drivers fail to be fully elucidated. In our study, evident seasonal and cyclical components were observed with the aid of the HP method; for example, every year from April until July, there was peak activity accounting for 59.581% of all notified cases, among which May and June were of particular concern, as they accounted for 60.952% of cases occurring in high-risk seasons. However, between January and February annually, there was a dramatic decline in the reported cases. A similar seasonal distribution was also revealed in recent years in other countries, including Singapore^41^, Malaysia^38^ and most regions of China^6,39,40,42^, containing Hong Kong, Taiwan, Shenzhen, Ningbo, Shandong, Zunyi, Guangdong and Guangzhou^6^. Moreover, outbreaks commonly occurred during the 4 months as well^42,43^. Additionally, two peaks could be noted in our data from June 2008 to June 2018, the first and stronger peak primarily occurred during the high-risk season, and the weaker peak was chiefly observed from August to November annually; the appearance of the two peaks was also reported in another study of southern China^6^. This seasonal pattern is consistent with that of Hong Kong^39^, Taiwan^40^ and Vietnam^37^. The single peak was customarily observed in northern China^44^, and earlier studies on the temporal characteristics of HFMD in Japan^3^ and Malaysia^38^ matched that in northern China. This may be pertinent to the different viruses, geographical differences or changing risk (e.g., school attendance, temperature, humidity or other meteorological drivers). Furthermore, in studies of particular regions of China, the leading agents (EV71 and CVA16) are also distributed in various peaks^45^, where the pathogenic agent EV71 is predominant in the stronger peak months. By contrast, CVA16 is more inclined to circulate in the more vulnerable population. These two etiologic factors are notably attenuated in January and February. Regarding the seasonal variations, climatic factors are possibly responsible for such a discrepancy (e.g., the ability of the causative agents to survive outside the host, the variability in the behavior and immune level of the host by climatic factors, and the inclination of people to go outdoors in summer rather than in winter increases the chances of person-to-person contact causing the etiologic agents to more easily achieve transmission among humans by virtue of spreading-factors)^6^.

To understand the epidemic situation in advance of the coming years, the constructed LSTM model with the best-fitting and best-predicting performance was adopted to calculate forecasts for the next two years. The results indicated that although the estimated observations would not show a large amplitude of oscillations relative to the in-sample data obtained, HFMD morbidity cases remained high, among which the highest-risk seasonality seemed to occur in June and July. Similar to prior findings^6^, two apparent seasonal peaks will be observed separately in subsequent Junes and Octobers, in all probability. Thus, due attention and instant action should be paid to these months and a response should be prompted, such as health promotion education; prevention at and control of key locations, particularly in nurseries and schools; vaccination and financial support. In addition, the prevention and control strategies for the rest of the low-risk months should fail to be ignored. In summary, the expected number of cases of HFMD remain present and still comparatively large, demonstrating that China is still afflicted with a chronic threat of HFMD.

Some limitations should be acknowledged in this work. First, no theoretical guidance can be adopted to identify the optimum number of hidden units, feedback delays and other key parameters during the establishment of ANN models. In practice, they are frequently selected by trial and error, and the specific forecasting process is poorly understood. Second, estimating the 95% uncertainty bounds for the predictions remains an additional problem. Third, the aggregated HFMD incidence case data utilized were obtained from nationwide passive infectious disease surveillance. We thus fail to rule out artifactual monitoring biases (e.g., substantial underreporting, misdiagnosis and delay). Fourth, the statistical predictions do not take known drivers into account and lack any epidemiological data other than case numbers and months, owing to their unavailability. Therefore, whether further studies that take these variables into account will have the potential to boost the fit and predictive ability remains to be authenticated. Fifth, albeit the LSTM technique built can be considered to be an instrumental tool for the medium-long-term estimation of future trends in HFMD incidence case data, in applications, this network is expected to be updated in due course with the incident cases to ensure its superiority in predictive performance. Sixth, detailed data on HFMD notifications are missing (e.g., age and sex), which precludes further analysis in the present work. Lastly, the LSTM model was developed based only on nationwide monitoring data over the period from 2008 to2018. These results therefore need to be interpreted with caution, and the analytic results can represent only entire epidemics of HFMD on the Chinese mainland. Remodeling for the region-specific notified HFMD cases time series may act as guidance for the formulation of targeted public health strategies, and whether the model is appropriate to calculate predictions for other kinds of communicable diseases requires further study.

In conclusion, notwithstanding its flaws, our study does indicate that the LSTM model established can provide more accurate predictions, be it in the in-sample dataset or the out-of-sample dataset. For the HFMD notified case time series compared with the individual SARIMA and NAR models, the LSTM model may be a beneficial tool for the early detection and advanced warning of HFMD activities in mainland China and can allow the official government to allocate health resources effectively and appropriately formulate the preventive and control planning for HFMD. Additionally, the number of forecasted incident cases are still relatively large and indeed present in the imminent future, this issue warrants to be resolved urgently and strategically within the effective measures taken.

## Materials and Methods

### Data collection

In this study, the aggregated monthly and yearly reported cases of HFMD, available from June 2008 to June 2018, were obtained from the notifiable infectious disease monitoring system provided by the Chinese Center for Disease Control and Prevention (CDC) (http://www.nhfpc.gov.cn/jkj/s3578/new_list.shtml). A total of 121 observations covering 11 years were collated and summarized. Subsequently, the whole dataset was split into two blocks to build the models, among which the first 109 data points (from June 2008 to June 2017) were regarded as in-sample modeling horizons, while the remaining 12 data points (from July 2017 to June 2018) were considered as out-of-sample predictive horizons. Since the sample length and time periods adopted to construct the models might have an impact on the forecasting power, two additional data categories were provided to test the robustness of the models developed, among which the first 103 (from June 2008 to December 2016) and 115 data points (from June 2008 to December 2017) were considered as in-sample modeling horizons, while the other 18 (from January 2017 to June 2018) and 6 data points (from January 2018 to June 2018) were used as out-of-sample predictive horizons.

In China, HFMD is clinically diagnosed by physicians, and the laboratory confirmed the diagnosis dependent on the detection of specific nucleic acids, the isolation of enterovirus related to pathogenic factors and the detection of a fourfold change in neutralizing antibodies. In addition, verified cases must be registered within 24 hours, and duplicate cases must be deleted by professionals at the end of the same month. Ethical approval or consent is not required for our present study owing to the public availability of HFMD surveillance data in China.

### Constructing the SARIMA model

The Box-Jenkins method of ARIMA(p, d, q) has been the most commonly used statistical forecasting technique for time series data that display no seasonality^46^. However, in applications, particularly in the morbidity time series of diseases, this time series frequently shows marked seasonal and cyclic tendencies^30^. Consequently, to avert losing significant series traits, a seasonal ARIMA method, specified as SARIMA(p, d, q)(P, D, Q)_s_, has been proposed to reveal data with those patterns^47^. In this model, the actual observation can be represented as a linear combination of the prior observation and the error sequence. As such, the secular change and seasonal variation of time series are captured in the SARIMA method as interpretable terms^48^. Although, of note, the linear SARIMA method can also model periodicity, the fitted cyclic change remains invariably symmetric. In our present study, considering the characteristics of HFMD incident case sequences containing evident cyclical and seasonality^6^, a typical SARIMA model will be a useful tool in predicting the future temporal trends^17^, among which the seasonal part of HFMD was taken for the predictors and the monthly HFMD incidence time series was used for the dependent variable. The final formula of a SARIMA model can be expressed as:

1 $$\{\begin{array}{c}\varphi (B){\rm{\Phi }}({B}^{{\rm{s}}}){{\rm{\Delta }}}^{d}{{\rm{\Delta }}}_{s}^{D}{X}_{t}=\theta (B){\rm{\Theta }}({B}^{s}){\varepsilon }_{t}\\ E({\varepsilon }_{t})=0,\,Var({\varepsilon }_{t})={\sigma }_{\varepsilon }^{2},E({\varepsilon }_{t}{\varepsilon }_{s})=0,s\ne t\\ E({X}_{s}{\varepsilon }_{t})=0,{\forall }_{s} < t\end{array}$$

where B refers to the backshift operator, ɛ_t_ denotes the errors from HFMD series, S signifies the length of seasonal cycle of HFMD notifications, d and D are the non-seasonal and seasonal differenced times, respectively. In the SARIMA model notation, p and q represent the orders of the non-seasonal autoregressive and moving average models, respectively; P and Q represent the orders of the seasonal autoregressive and moving average models, respectively. ${\nabla }^{{\rm{d}}}={(1-B)}^{d}$, ${\nabla }_{S}^{D}={(1-B)}^{SD}$, $\varphi (B)=1-{\varphi }_{1}B-\cdot \cdot \cdot \,-\,{\varphi }_{p}{B}^{p}$,$\theta (B)=1-{\theta }_{1}B-\cdot \cdot \cdot -{\theta }_{q}{B}^{q}$, ${\rm{\Phi }}({B}^{s})=1-{{\rm{\Phi }}}_{1}{B}^{s}-\cdot \cdot \cdot -{{\rm{\Phi }}}_{P}{B}^{P{\rm{s}}}$, ${\rm{\Theta }}({B}^{s})=1-{{\rm{\Theta }}}_{1}{B}^{s}-\cdot \cdot \cdot -{{\rm{\Theta }}}_{Q}{B}^{Q{\rm{s}}}$.

We utilized the R statistical package (version 3.4.3, R Development Core Team, Vienna, Austria) and SPSS software (version 17.0, IBM Corp, Armonk, NY) to construct the SARIMA model. The development of the SARIMA approach is under the assumption of a stationary incidence time series^10^. Therefore, in this research, the ADF test was used to identify whether the actual reported cases and processed data using differencing or a transformation technique accomplished stationarity^49^. Afterwards, the autocorrelation function (ACF) and partial autocorrelation function (PACF) plots, the Schwarz Bayesian criterion (SBC), the Akaike information criterion (AIC) and the corrected Akaike Information criterion (AICc), along with the Lagrangian multiplier (LM) and Ljung-Box *Q* tests were applied to estimate and diagnose the model^50^. The above mentioned modeling procedures were repeatedly conducted until the best-performing model was ultimately discovered.

### Establishing the NAR model

Complexities and challenges in understanding the temporal characteristics of infectious diseases that exist are the complicated nonlinear interactions among different dimensions in real-world scenarios^16^. Artificial neural networks (ANNs) can adequately enable arbitrarily intricate non-stationary series to attain any desired accuracy owing to their powerful flexible nonlinear mapping capacity, and they have been considered as a function approximator applied in the domains of environmental forecasting, electrical energy and medicine^10,16^. The NAR model is a leading shallow, dynamic recurrent neural network (RNN) that is based on the linear autoregressive model with the ability to time-varying the state of interconnected neurons^51^, can be adopted to explore the nonlinear relationship between the response variable and its predictors owing to its network architecture with a hidden layer accompanied by a sigmoid transfer function that allows it to have no restrictions on the parameters that comply with the requirement of stationarity^52–54^. Furthermore, with the aid of tapped delay lines, the NAR technique also has a short-term memory function for the previous inputs and outputs, which makes its response at any given time rely not only on the present inputs but on the history of the inputs series as well^55^. For a time series with obvious seasonality, when providing suitable observations from the same season as inputs, this network can capture the time series components of periodicity, seasonality and secular trend adequately well with respect to the appropriate inputs that require a multitude of experiments to discover the optimum. Accordingly, this method can provide reliable forecasts for current HFMD incidence case series including linear and nonlinear information. The specified equation of the NAR method can be written as:

2 $$y(t)=f(y(t-1),\,y(t-2),\,\cdot \cdot \cdot ,\,y(t-d)),$$

where y(t) represents the predicted points of the HFMD incidence series relied merely on the prior data of lagged period *d*.

In this work, the graphical user interface (GUI) in MATLAB (Version R2014a, MathWorks, Natick, MA, USA) was employed to automatically create an advanced script prior to modeling an NAR. First, the actual HFMD observations were processed between 0 and 1 using a normalized approach^56^ to facilitate further analysis. Second, the *dividerand* function was used to randomly divide the in-sample data into training, validation and testing subsets, following ratios of 70%, 15% and 15%, respectively. In the robustness-test data, the abovementioned ratio in the training, validation and testing subsets was also used in the first test dataset, while another commonly used ratio of 80%, 10% and 10% corresponding to the training, validation and testing subsets, respectively, was employed in the second test dataset. Third, the number of hidden neurons and delays *d* were adjusted by repeated attempts with the Levenberg-Marquardt algorithm in an open feedback loop. The response plot of outputs and targets, correlograms and input-error cross-correlation plots, coupled with the mean square error (MSE) and correlation coefficient (R), were offered to choose the best-fitting model. Finally, the training open-loop form was transformed to a closed loop to achieve a goal of multistep-ahead forecasting (Supplementary Fig. S35).

### Establishing the LSTM model

As mentioned above, coincident with the increase in the time lag has been a decrease in the long-term learning ability during the training of an NAR method due to a vanishing or exploding gradient problem, which is a major flaw for NAR method forecasting^26^. A LSTM model, not the least prevalent and rewarding variant of the conventional RNNs, can overcome this disadvantage encountered in an NAR model as it is capable of maintaining state and identifying traits over the length of the sequences used^26,27^. The LSTM technique has a special layered architecture with memory blocks that contain one or more self-connected memory cells^22,57^, surrounded by three gating units, including the input, output and forget gates, that can continuously perform write, read and reset operations, to preserve information (Supplementary Fig. S36)^57,58^. Such a configuration can keep the states persisting or communicating between updates of the weights with the progress of each epoch; moreover, it can reinforce the RNN by capturing the long-term dynamics of the time series components of periodicity, seasonality and secular trend in addition to the short-term dynamics. The estimated equations of the LSTM model can be defined as:

3 $${i}_{t}=\sigma ({W}_{xi}{x}_{t}+{W}_{hi}{h}_{t-1}+{W}_{ci}{c}_{t-1}+b{}_{i})$$

4 $${f}_{t}=\sigma ({W}_{xf}{x}_{t}+{W}_{hf}{h}_{t-1}+{W}_{cf}{c}_{t-1}+b{}_{f})$$

5 $${c}_{t}={f}_{t}{c}_{t-1}+{i}_{t}\,\tanh ({W}_{xc}{x}_{t}+{W}_{hc}{h}_{t-1}+{W}_{ci}{c}_{t-1}+b{}_{c})$$

6 $${o}_{t}=\sigma ({W}_{xo}{x}_{t}+{W}_{ho}{h}_{t-1}+{W}_{co}{c}_{t-1}+b{}_{o})$$

7 $${h}_{t}={o}_{t}\,\tanh ({c}_{t})$$

8 $$y(t)={W}_{yh}(f({W}_{hx}y(t-1)+{W}_{hh}{h}_{t-1}+{b}_{h})+{b}_{y}$$

where *i*_*t*_ refers to the input gate; *f*_*t*_ is the forget gate; *c*_*t*_ stands for the states of memory cell at time *t*; *o*_*t*_ represents the output gate; *h*_*t*_ is the hidden states at time *t*; *W*_*xc*_, *W*_*xi*_, *W*_*xf*_ and *W*_*xo*_ are the weight matrices connecting the input signals; *x*_*t*_, *W*_*hc*_, *W*_*hi*_, *W*_*hf*_ and *W*_*ho*_ represent the weight matrices connecting the hidden layer output signals; *h*_*t*_, *W*_c*i*_, *W*_*cf*_ and *W*_*co*_ stand for the diagonal matrices connecting the neuron activation functions; *b*_*i*_, *b*_*c*_, *b*_*f*_, *b*_*o*_, *b*_*h*_ and *b*_*y*_ refer to the bias vectors; *σ* is the activation function (tanh or sigmoid); y(t) is the predicted points of the HFMD incidence series; *W*_*h*x_, *W*_*h*h_, and *W*_*yh*_ are the input-hidden weight matrix, hidden-hidden weight matrix and hidden-output weight matrix, respectively; and ${y}_{t-1}=({y}_{t-1,}{y}_{t-2,\cdots ,}{y}_{t-d})^{\prime}$ is a vector including time steps of the series.

Our prediction project using the LSTM model for regression was conducted with Keras. First, the actual HFMD notifications were rescaled between 0 and 1 using a normalized preprocessing approach. Second, in-sample data were separated into two blocks, 75% of them would be used for the training model, whereas the remaining 25% of the data would be utilized to validate the generalization and to simulate performance. Similarly, in the robustness-test data, the same data classification described above was applied to the first test dataset, while 70% and 30% data points were taken as training and validation subsets, respectively, out of the second test dataset. Third, the back propagation through time (BPTT) algorithm^59^ was adopted for LSTM training with various time steps, and hidden layer neurons used to select the preferred model relied on the minimum root mean square error loss and satisfactory ACF plot^22^. Finally, the best-fitting architecture was chosen to generate out-of-sample predictions, and then the results should further be transformed to the simulated and forecasted values from the original observations with the inverse transform technique.

### Measuring for accuracy

In order to distinguish the stimulation and forecasting accuracies from the selected various models, the root mean square error (RMSE), mean absolute error (MAE) and mean absolute percentage error (MAPE) were ultimately adopted to measure the performance accuracy.

9 $${\rm{RMSE}}=\sqrt{\frac{1}{N}\sum _{i=1}^{N}{({X}_{i}-{\bar{X}}_{i})}^{{\rm{2}}}}$$

10 $${\rm{MAE}}=\frac{1}{N}\sum _{i=1}^{N}|{X}_{i}-{\bar{X}}_{i}|$$

11 $${\rm{MAPE}}=\frac{1}{N}\sum _{i=1}^{N}\frac{|{X}_{i}-{\bar{X}}_{i}|}{{X}_{i}}$$

Where *X*_*i*_ stands for the actual notified notifications, ${\bar{X}}_{i}$ represents the simulated and predictive values with the selected preferred methods, *N* is the number of simulations and predictions under the models used.

## Supplementary information

Supplementary Materials

Table S12

## Data Availability

These data can be extracted as presented in the website of data collection or please contact the first author on reasonable request.
