# Supplementary material for: Development and evaluation of a deep learning approach for modeling seasonality and trends in hand-foot-mouth disease incidence in mainland China
Source: Sci Rep. 2019 May 29;9:8046. doi: 10.1038/s41598-019-44469-9 (PMC6541597; doi:10.1038/s41598-019-44469-9)
Supplement: Supplementary file 1 — Supplementary Materials [file 41598_2019_44469_MOESM1_ESM.pdf]

Supplementary information: Development and evaluation of a deep learning approach for modeling seasonality and trends in hand-foot-mouth disease incidence in mainland China

Yongbin Wang[1,+], Chunjie Xu[2,+], Shengkui Zhang[1], Li Yang[1], Zhende Wang[1], Ying Zhu[1], Juxiang Yuan[1,*]

[1] School of Public Health, North China University of Science and Technology, Tangshan, Hebei Province, P.R. China; 568019636@qq.com(Y.B.W.); 1527317477@qq.com(S.K.Z.); 593177072@qq.com(L.Y.); 9002629472@qq.com (Z.D.W.); 121545724@qq.com(Y.Z.)

[2] School of Public Health, Capital Medical University, Beijing 100069, P.R. China; 2248209559@qq.com(C.J.X.)

* Corresponding author: Juxiang Yuan, No.21 Bohai Road, Caofeidian Xincheng, Tangshan City, Hebei Province, 063210, P.R. China; Tel: +86-315-8805002; e-mail: yuanjx@ncst.edu.cn

[+] These authors contributed equally to this work

Supplementary Figure and Table captions

Figure S1. Systematic seasonal factors for HFMD notified cases at national level from June 2008 to June 2018 using the decomposition method. It can be seen that the reported cases HFMD series shows an apparent seasonality with the peak activities in April until July annually.

Figure S2. ACF and PACF graph with the first-order non-seasonal difference(d=1) of monthly HFMD incidence series in mainland China from June 2008 to June 2017. This plot suggests that after the first-order non-seasonal difference, the time-varying trend tends to stabilize approximately and there is a marked seasonal pattern owing to the local maximum values at lags 12, 24 and 36 in the ACF plot.

Figure S3. ACF and PACF graphs with the first-order seasonal difference(D=1) of monthly HFMD incidence series in mainland China from June 2008 to June 2017. Based on the plot, we can conclude that the differenced series has successfully been stationarity.

Figure S4. The Q-Q plot of residuals from SARIMA(1,1,2)$(1,1,0)_{12}$ model for HFMD series from June 2008 to June 2017. This plot suggests that the distribution of produced residuals may have a tail thicker than that of a normal distribution.

Figure S5. Autocorrelation function(ACF) and partial autocorrelation function(PACF) graphs the first-order non-seasonal difference(d=1) of monthly HFMD incidence series in mainland China from June 2008 to December 2016. The plot shows that the differenced series looks much more stationary when compared with the original time series. Nonetheless there still is a marked seasonal pattern in this differenced series.

Figure S6. Autocorrelation function(ACF) and partial autocorrelation function(PACF) graphs with the first-order seasonal difference(D=1) of monthly HFMD incidence series in mainland China from

June 2008 to December 2016. Based on the plot, we may well consider the seasonal differenced series as a stationary series.

Figure S7. The resultant plots of fit goodness tests from SARIMA(1,0,1)(1,1,1)$_{12}$ model for HFMD notified cases series from June 2008 to December 2016. (a) Standardized residuals. (b) Autocorrelation function (ACF) graph of errors across varying lag times. None of the autocorrelation coefficients are out of the 95% confidence intervals in this residual series. (c) Partial autocorrelation function (PACF) graph of errors. (d) Q-statistic *P*-values. There are large *P* values at the significance level of 5%. Diagnostic checking indicates the chosen SARIMA specification can provide a reasonable approximation to the HFMD notified cases series from June 2008 to December 2016.

Figure S8. The Q-Q plot of residuals from SARIMA(1,0,1)(1,1,1)$_{12}$ model for HFMD series from June 2008 to December 2016. This plot suggests that the distribution of produced residuals has a tail thicker than that of a normal distribution.

Figure S9. Autocorrelation function(ACF) and partial autocorrelation function(PACF) graphs the first-order non-seasonal difference(d=1) of monthly HFMD incidence series in mainland China from June 2008 to December 2017. The plot shows that the differenced series looks much more stationary when compared with the original time series. Nonetheless there still is an obvious seasonal pattern in this differenced series.

Figure S10. Autocorrelation function(ACF) and partial autocorrelation function(PACF) graphs with the first-order seasonal difference(D=1) of monthly HFMD incidence series in mainland China from June 2008 to December 2017. On the basis of the plot, we can observe that this differenced series meets the need of modeling for SARIMA method.

Figure S11. The resultant plots of fit goodness tests from SARIMA(1,0,1)(1,1,1)$_{12}$ model for HFMD notified cases series from June 2008 to December 2017. (a) Standardized residuals. (b) Autocorrelation function (ACF) graph of errors across varying lag times. The spikes all fall within the 95% confidence intervals in this residual series. (c) Partial autocorrelation function (PACF) graph of errors. (d) *P* values for Ljung-Box statistic.. There are large *P* values at the significance level of 5%. Diagnostic checking indicates the chosen SARIMA specification is suitable for the HFMD notified cases series from June 2008 to December 2017.

Figure S12. The Q-Q plot of residuals from SARIMA(1,0,1)(1,1,1)$_{12}$ model for HFMD series from June 2008 to December 2017. This plot suggests that residuals are departure from normality at the tails.

Figure S13. The Q-Q plot of residuals from NAR model for HFMD series from June 2008 to June 2017. The Q-Q plot of the residuals shows departure from normality at the tails.

Figure S14. The regression plots for the NAR model outputs with respect to targets for training, validation, and test in the dataset from June 2008 to June 2017.

Figure S15. The layer architecture of NAR model for the HFMD notified cases series from June 2008 to December 2016. (A) The opened loop mode; (B) The closed loop mode. This NAR model is comprised of a hidden layer with 17 neurons and 5 delays and an output layer with 1 neuron. The model adopts tapped delay lines to store prior data of the x(t) and y(t) series as well. Among which, the output results of the model, y(t), is fed back to the input (through delays), since y(t) is a function of y(t – 1), y(t – 2), ...,y(t – d). Nevertheless, in order to train more efficiently, the training can be undertaken in open loop. After training, then the opened loop mode should be transformed to the closed loop mode for multistep-ahead forecasting.

Figure S16. The resulting plots of fit goodness tests from the best-fitting NAR model for HFMD notified cases series from June 2008 to December 2016. (**a**) Standardized residuals. (**b**) Autocorrelation function (ACF) plot of errors across varying lag times. All of the autocorrelations fail to be beyond the estimated 95% uncertainty bounds around zero across varying lag times apart from the one from ACF plot at zero lag that should occur and also occurring at lag 11, we should not be surprised as this can easily happen by chance alone. Overall, the plot manifests that the network appears to have captured the dependence hidden behind the HFMD notified cases series. (**c**) Input-to-error correlation plot for varying lags. The input-error cross-correlation function illustrates how the residuals are interrelated with the series of x(t). All of the correlations fall within the confidence bounds around zero, which hints the developed model is a perfect specification. (**d**) Q-statistic *P*-values. Analyses form the plots demonstrate the constructed model is adequate in excavating the information of this time series.

Figure S17. The response of output and target for HFMD time series from June 2008 to December 2016 at various time points. This plot exhibits which time points are elected as the training, validation and testing subsets, along with their corresponding errors between inputs and targets. In view of the small errors, a further suggestion that the fitting is fairly accurate.

Figure S18. The regression plots for the best-fitting NAR model outputs with respect to targets for training, validation, and test in the dataset from June 2008 to December 2016.

Figure S19. The Q-Q plot of residuals from NAR model for HFMD series from June 2008 to December 2016. The Q-Q plot of the residuals shows marked departure from normality at the tails.

Figure S20. The layer architecture of NAR model for HFMD notified cases series from June 2008 to December 2017. (A) The opened loop mode; (B) The closed loop mode. This NAR model is

comprised of a hidden layer with 19 neurons and 6 delays and an output layer with 1 neuron. The model adopts tapped delay lines to store prior data of the x(t) and y(t) series as well. Among which, the output results of the model, y(t), is fed back to the input (through delays), since y(t) is a function of y(t – 1), y(t – 2), ...,y(t – d). Nevertheless, in order to train more efficiently, the training can be undertaken in open loop. After training, then the opened loop mode should be transformed to the closed loop mode for multistep-ahead forecasting.

Figure 21. The resulting plots of fit goodness tests from the best-fitting NAR model for HFMD notified cases series from June 2008 to December 2017. (**a**) Standardized residuals. (**b**) Autocorrelation function (ACF) plot of errors across varying lag times. All of the autocorrelations fail to be beyond the estimated 95% uncertainty bounds around zero across varying lag times apart from the one from ACF plot at zero lag that should occur. The plot manifests that the network appears to have captured the dependence hidden behind the HFMD notified cases series. (**c**) Input-to-error correlation plot for varying lags. The input-error cross-correlation function illustrates how the residuals are interrelated with the series of x(t). All of the correlations fall within the confidence bounds around zero, which hints the developed model is a perfect specification. (**d**) Q-statistic *P*-values. Analyses form the plots demonstrate the constructed model is adequate in excavating the information of this time series.

Figure S22. The response of output and target for HFMD time series from June 2008 to December 2017 at various time points. This plot exhibits which time points are elected as the training, validation and testing subsets, along with their corresponding errors between inputs and targets. In view of the small errors nearly lying between -0.2 and 0.2, a further suggestion that the fitting is fairly accurate.

Figure S23. The regression plots for the best-fitting NAR model outputs with respect to targets for training, validation, and test in the dataset from June 2008 to December 2017.

Figure S24. The Q-Q plot of residuals from the best-simulating NAR model for HFMD series from June 2008 to December 2017. This plot suggests that the distribution of produced residuals has a tail thicker than that of a normal distribution.

Figure S25. The regression plots for the LSTM model outputs with respect to targets for training and validation in the dataset from June 2008 to June 2017.

Figure S26. The Q-Q plot of residuals from LSTM model for HFMD series from June 2008 to June 2017. As exhibited in the plot of the normal Q-Q plot, the residuals approximately fall along the line. Thus the best-fitting LSTM model improves the normality dramatically compared with the basic NAR and SARIMA methods.

Figure S27. The training and validation performances for LSTM model at 300 epochs for the HFMD notified cases series from June 2008 to December 2016. This plot documents that the test set error and the validation set error have similar characteristics and no significant overfitting has occurred by iteration 300.

Figure S28. The resulting plots of fit goodness tests from the best-performing LSTM model for HFMD notified cases series from June 2008 to December 2016. (**a**) Standardized residuals. (**b**) Autocorrelation function (ACF) plot of errors across varying lag times. The ACF plot of forecasted errors reveals no individually evident autocorrelation at varying lags except for the one from ACF plot at zero lag that should occur. (**c**) Partial autocorrelation function (PACF) plot of residuals. (**d**) Q-statistic *P*-values. As shown, All *P*-values are larger than 0.05. These diagnostics manifest that the network is well suited to the dataset.

Figure S29. The regression plots for the best-presenting LSTM model outputs with respect to targets for training and validation in the dataset from June 2008 to December 2016.

Figure S30. The Q-Q plot of residuals from LSTM model for HFMD series from June 2008 to December 2016. As exhibited in the plot of the normal Q-Q plot, the points seem to follow the straight line fairly closely. This graph would not lead us to reject normality of the error terms in this model. Thus the best-fitting LSTM model improves the normality dramatically compared with the basic NAR and SARIMA methods.

Figure S31. The training and validation performances for LSTM model at 300 epochs for the HFMD notified cases series from June 2008 to December 2017. This plot documents that the test set error and the validation set error have similar characteristics and no significant overfitting has occurred by iteration 300.

Figure S32. The resulting plots of fit goodness tests from the LSTM model for HFMD notified cases series from June 2008 to December 2017. (**a**) Standardized residuals. (**b**) Autocorrelation function (ACF) plot of errors across varying lag times. The ACF plot of forecasted errors reveals no individually evident autocorrelation at varying lags except for the two points occurring at lags 2 and 15. For these two lagged points out of the estimated 95% confidence limit, they are also reasonable as this phenomenon can easily happen by chance alone. (**c**) Partial autocorrelation function (PACF) plot of residuals. (**d**) Q-statistic *P*-values. As shown, All *P*-values are larger than 0.05. These diagnostics manifest that the network is well suited to the dataset.

Figure S33. The regression plots for the best-presenting LSTM model outputs with respect to targets for training and validation in the dataset from June 2008 to December 2017.

Figure S34. The Q-Q plot of residuals from LSTM model for HFMD series from June 2008 to December 2017. The Q-Q plot suggests that the distribution of errors may have a tail thicker than that of a normal distribution and may be somewhat skewed to the right. However, In comparison

with the best-mimicking NAR and SARIMA approaches, the best-fitting LSTM model can improve the normality dramatically.

Figure S35. The layer architecture of NAR model for the HFMD notified cases series from June 2008 to June 2017. (A) The opened loop mode; (B) The closed loop mode. This NAR model is comprised of a hidden layer with 18 neurons and 5 delays and an output layer with 1 neuron. The model adopts tapped delay lines to store prior data of the x(t) and y(t) series as well. Among which, the output results of the model, y(t), is fed back to the input (through delays), since y(t) is a function of y(t – 1), y(t – 2), ...,y(t – d). Nevertheless, in order to train more efficiently, the training can be undertaken in open loop. After training, then the opened loop mode should be transformed to the closed loop mode for multistep-ahead forecasting.

Figure S36. The layer architecture of LSTM model. Above-mentioned these gates represent nonlinear summation units that gather activations from inside and outside the block, and dominate the activation of the cell via multiplications (small black circles). The input and output gates multiply the cell’s input and output when the forget gate multiplies the earlier state of the cell. The gate activation function ‘f’ ordinarily refers to the logistic sigmoid, which can limit the gate activations into [0, 1] intervals. The cell input and output activation functions (‘g’ and ‘h’) customarily stand for tanh or logistic sigmoid. In this layer architecture, dashed lines are the weighted join points from the cell to the gates, the remainder of lines within the block denote the unweighted join points. The only outputs from the block to the remaining the network emanate from the output gate multiplcication.

Table S1. The estimated parameters and performance indexes for the selected candidate models based on the original observations from June 2008 to June 2017.

Table S2. Estimated parameters of the SARIMA(1,0,1)(1,1,1)$_{12}$ model for the target series from June 2008 to December 2016.

Table S3. The goodness of fit test of the preferred SARIMA model for the target series from June 2008 to December 2016.

Table S4. Ljung-Box *Q* test of the residuals for the selected three optimal models fitted to the notified HFMD cases series from June 2008 to December 2016 at different lags.

Table S5. ARCH effect of the observations and residuals of the selected three models fitted to the notified HFMD cases series from June 2008 to December 2016 with LM test at various lags.

Table S6. Estimated parameters of the SARIMA(1,0,1)(1,1,1)$_{12}$ model for the target series from June 2008 to December 2017.

Table S7. The goodness of fit test of the preferred SARIMA model for target series from June 2008 to December 2017.

Table S8. Ljung-Box *Q* test of the residuals for the selected three optimal models fitted to the notified HFMD cases series from June 2008 to December 2017 at different lags.

Table S9. ARCH effect of the observations and residuals of the selected three models fitted to the notified HFMD cases series from June 2008 to December 2017 with LM test at various lags.

Table S10. The preferred NAR models' parameters of various target series.

Table S11. The preferred LSTM models' parameters of various target series.

Table S12. Future one hundred possible sample paths for the HFMD notified data in mainland China. This Table was provided in a Microsoft Excel.xlsx version on account of the plethora of data.

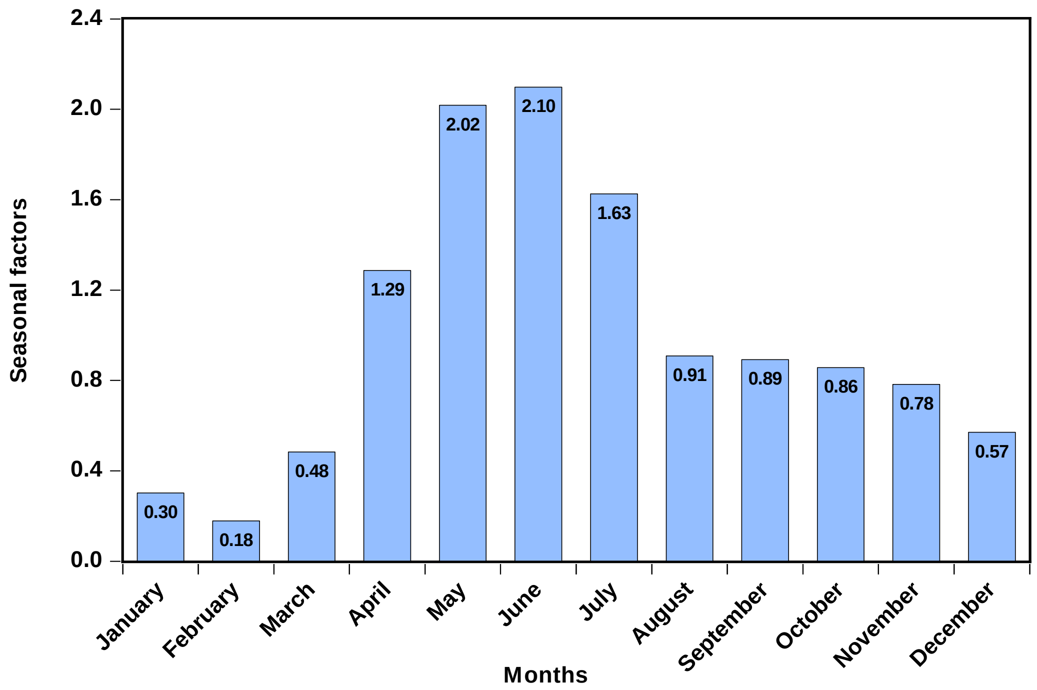

Figure S1. Systematic seasonal factors for HFMD notified cases at national level from June 2008 to June 2018 using the decomposition method. It can be seen that the reported cases HFMD series shows an apparent seasonality with the peak activities in April until July annually.

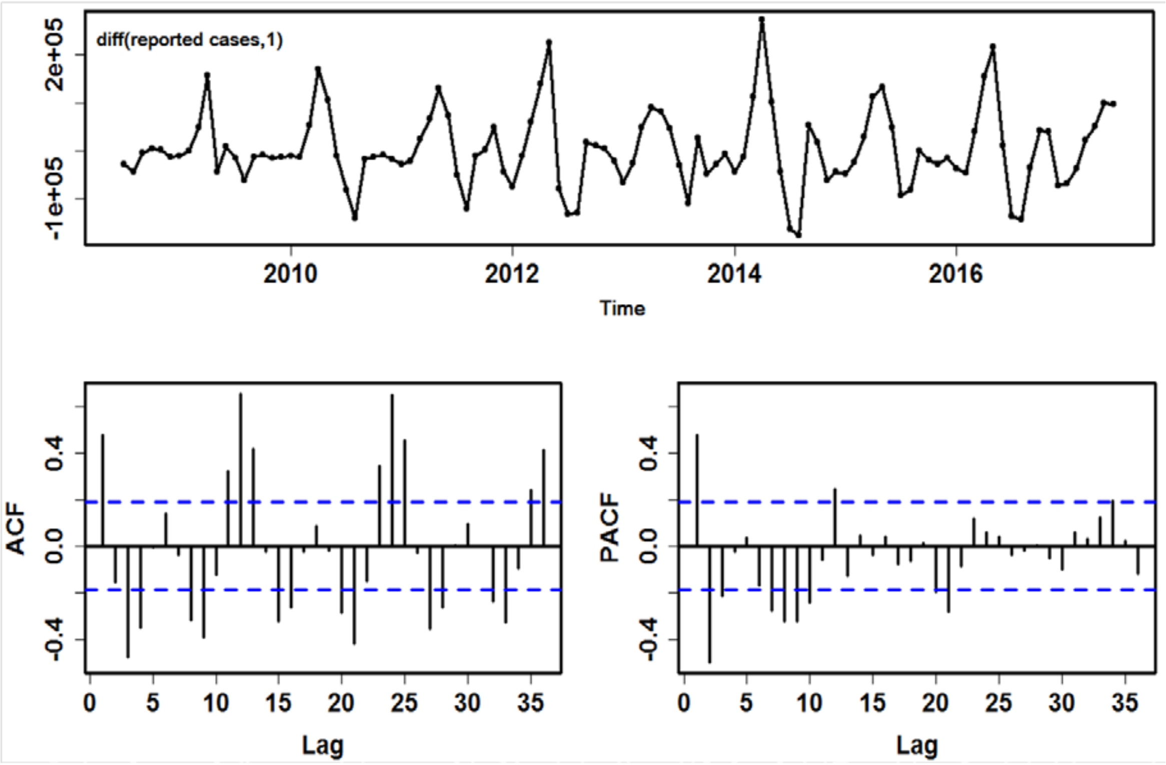

Figure S2. ACF and PACF graph with the first-order non-seasonal difference(d=1) of monthly HFMD incidence series in mainland China from June 2008 to June 2017. This plot suggests that after the first-order non-seasonal difference, the time-varying trend tends to stabilize approximately and there is a marked seasonal pattern owing to the local maximum values at lags 12, 24 and 36 in the ACF plot.

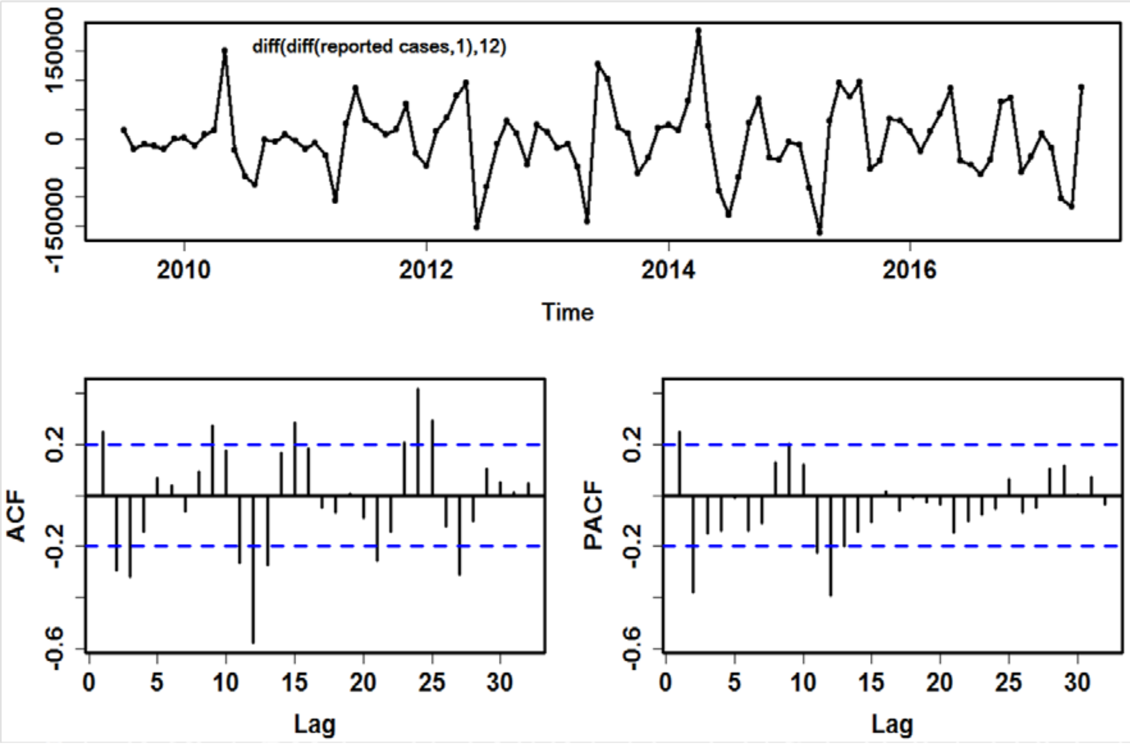

Figure S3. ACF and PACF graphs with the first-order seasonal difference(D=1) of monthly HFMD incidence series in mainland China from June 2008 to June 2017. Based on the plot, we can conclude that the differenced series has successfully been stationarity.

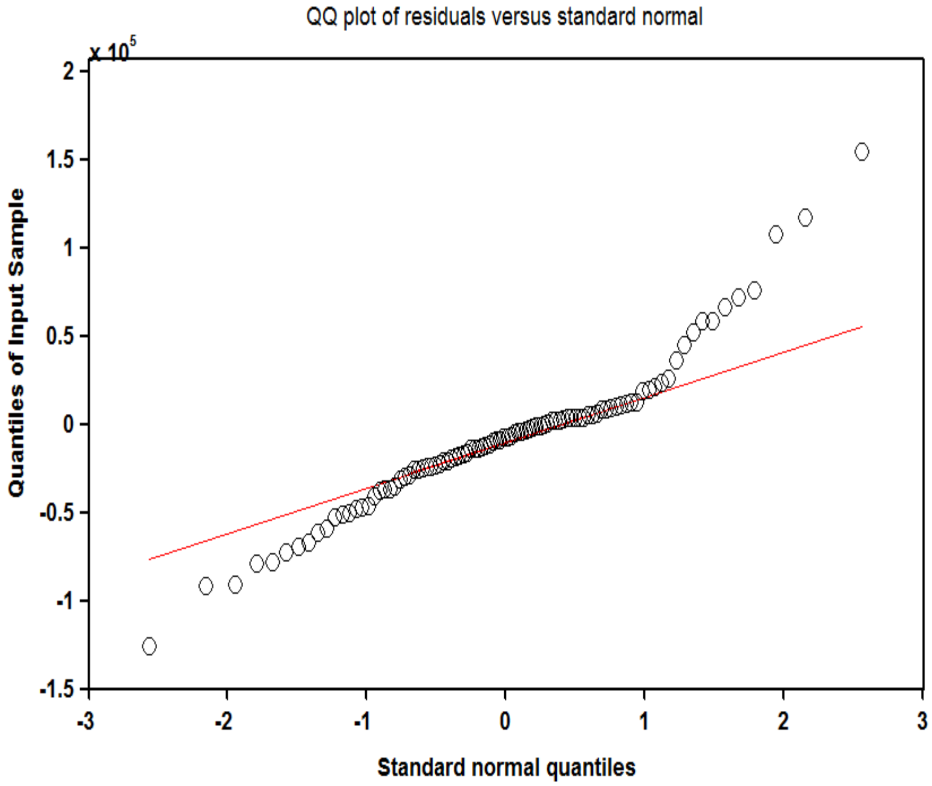

Figure S4. The Q-Q plot of residuals from SARIMA(1,1,2)(1,1,0)$_{12}$ model for HFMD series from June 2008 to June 2017. This plot suggests that the distribution of produced residuals may have a tail thicker than that of a normal distribution.

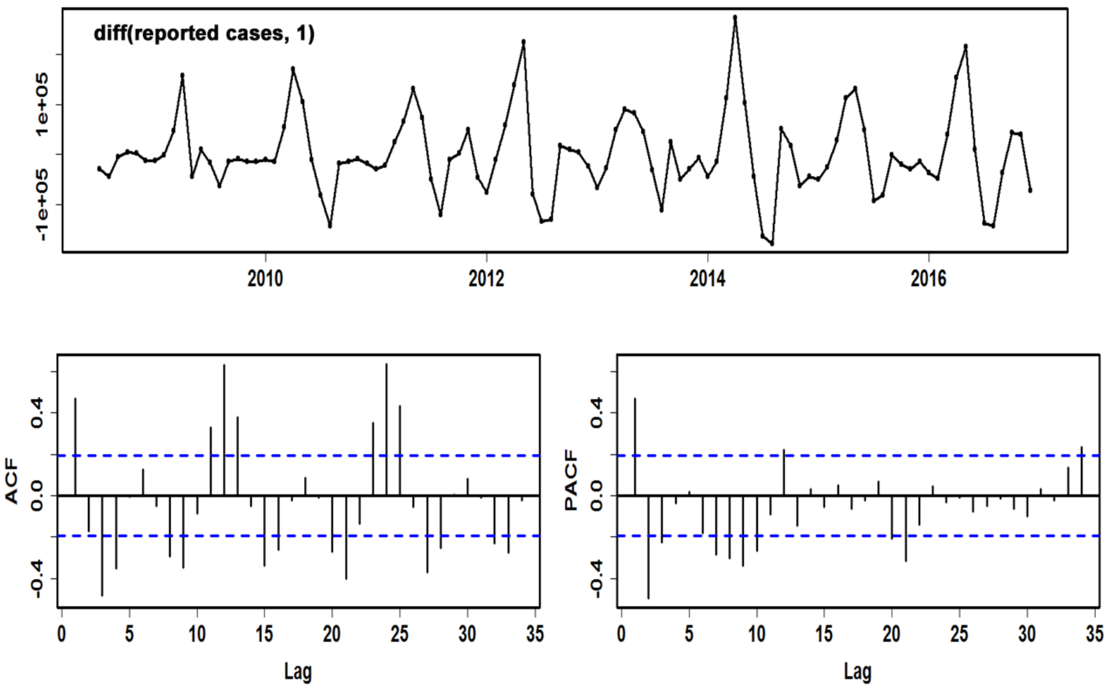

Figure S5. Autocorrelation function(ACF) and partial autocorrelation function(PACF) graphs the first-order non-seasonal difference(d=1) of monthly HFMD incidence series in mainland China from June 2008 to December 2016. The plot shows that the differenced series looks much more stationary when compared with the original time series. Nonetheless there still is a marked seasonal pattern in this differenced series.

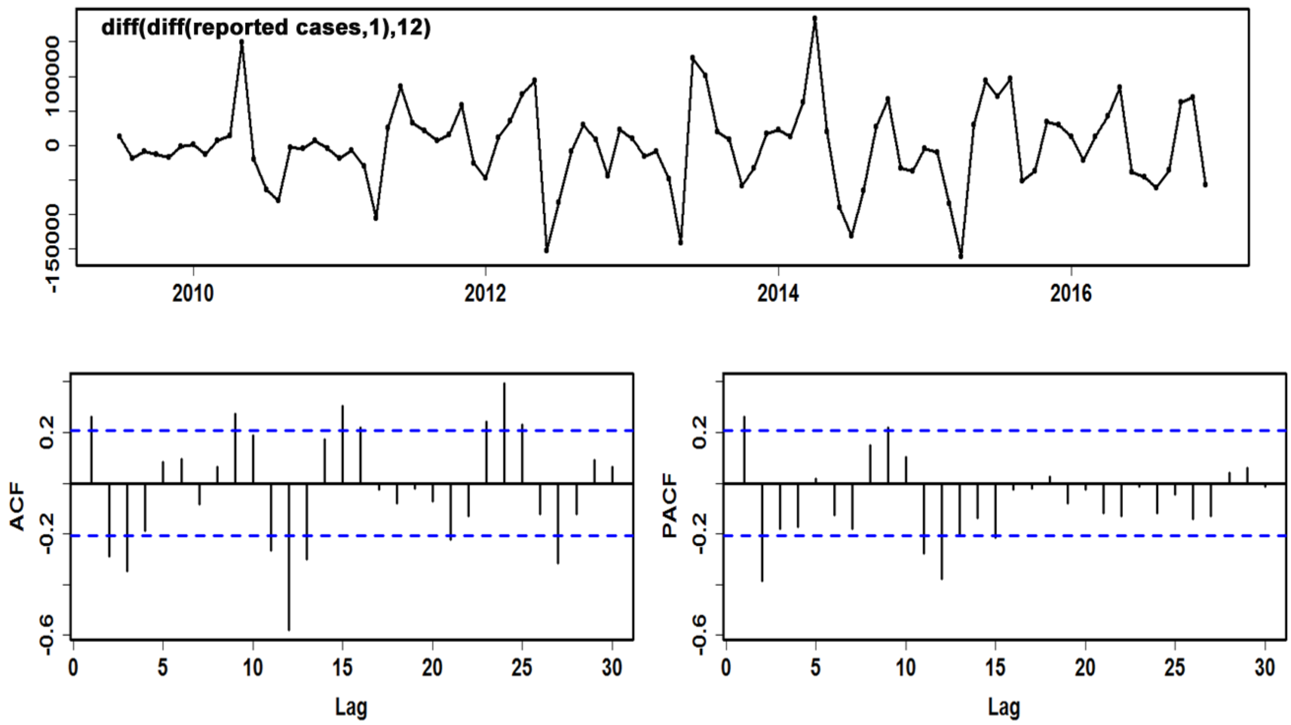

Figure S6. Autocorrelation function(ACF) and partial autocorrelation function(PACF) graphs with the first-order seasonal difference(D=1) of monthly HFMD incidence series in mainland China from June 2008 to December 2016. Based on the plot, we may well consider the seasonal differenced series as a stationary series.

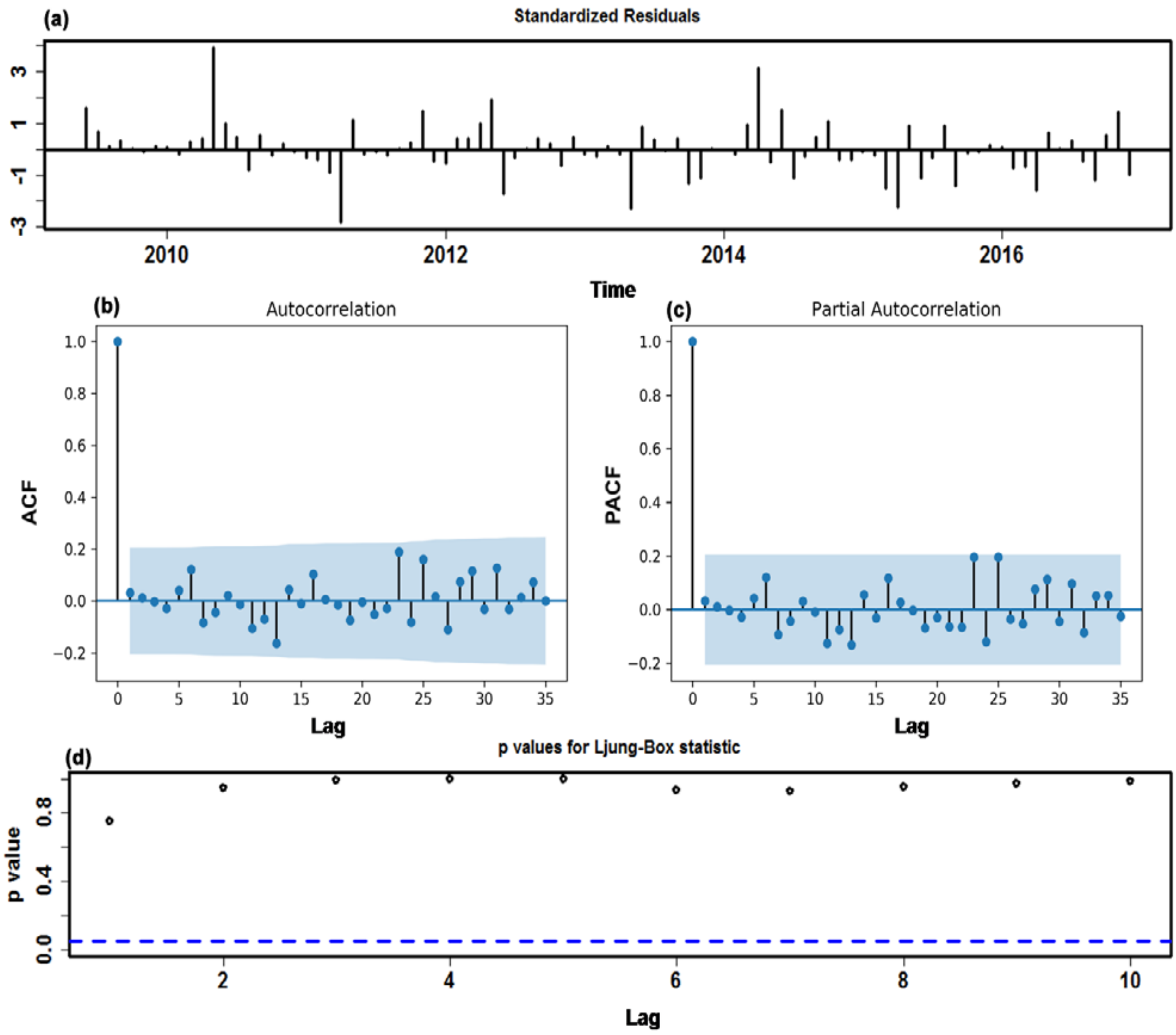

Figure S7. The resultant plots of fit goodness tests from SARIMA(1,0,1)(1,1,1)$_{12}$ model for HFMD notified cases series from June 2008 to December 2016. (a) Standardized residuals. (b) Autocorrelation function (ACF) graph of errors across varying lag times. None of the autocorrelation coefficients are out of the 95% confidence intervals in this residual series. (c) Partial autocorrelation function (PACF) graph of errors. (d) Q-statistic *P*-values. There are large *P* values at the significance level of 5%. Diagnostic checking indicates the chosen SARIMA specification can provide a reasonable approximation to the HFMD notified cases series from June 2008 to December 2016.

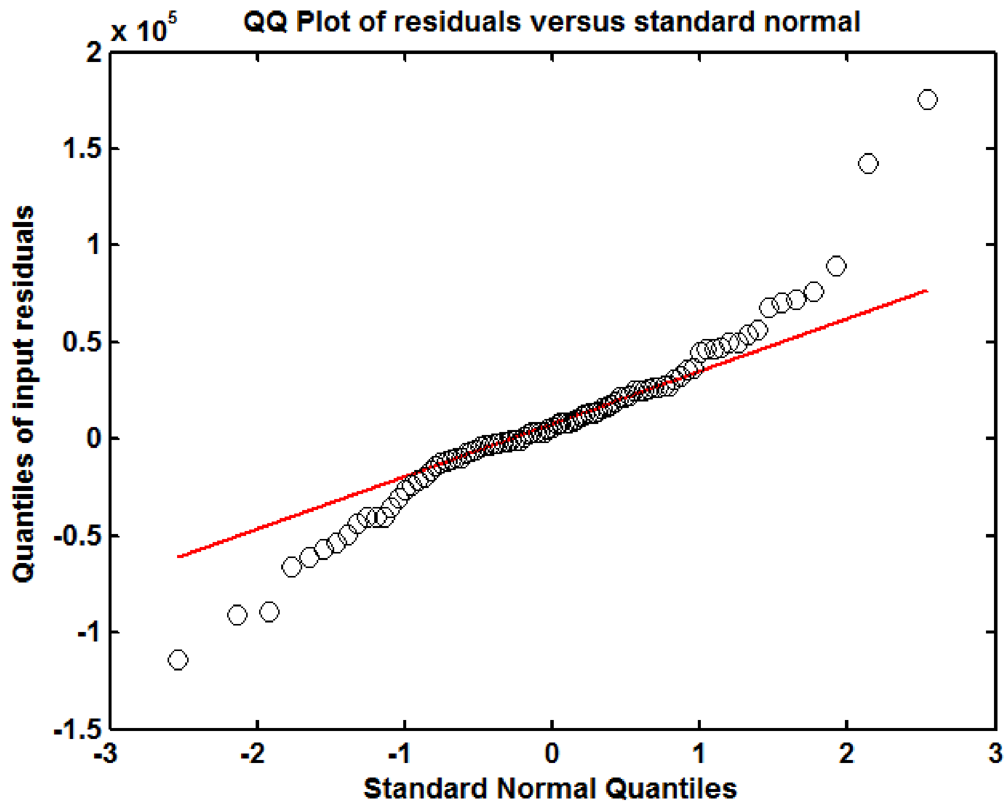

Figure S8. The Q-Q plot of residuals from SARIMA(1,0,1)(1,1,1)$_{12}$ model for HFMD series from June 2008 to December 2016. This plot suggests that the distribution of produced residuals has a tail thicker than that of a normal distribution.

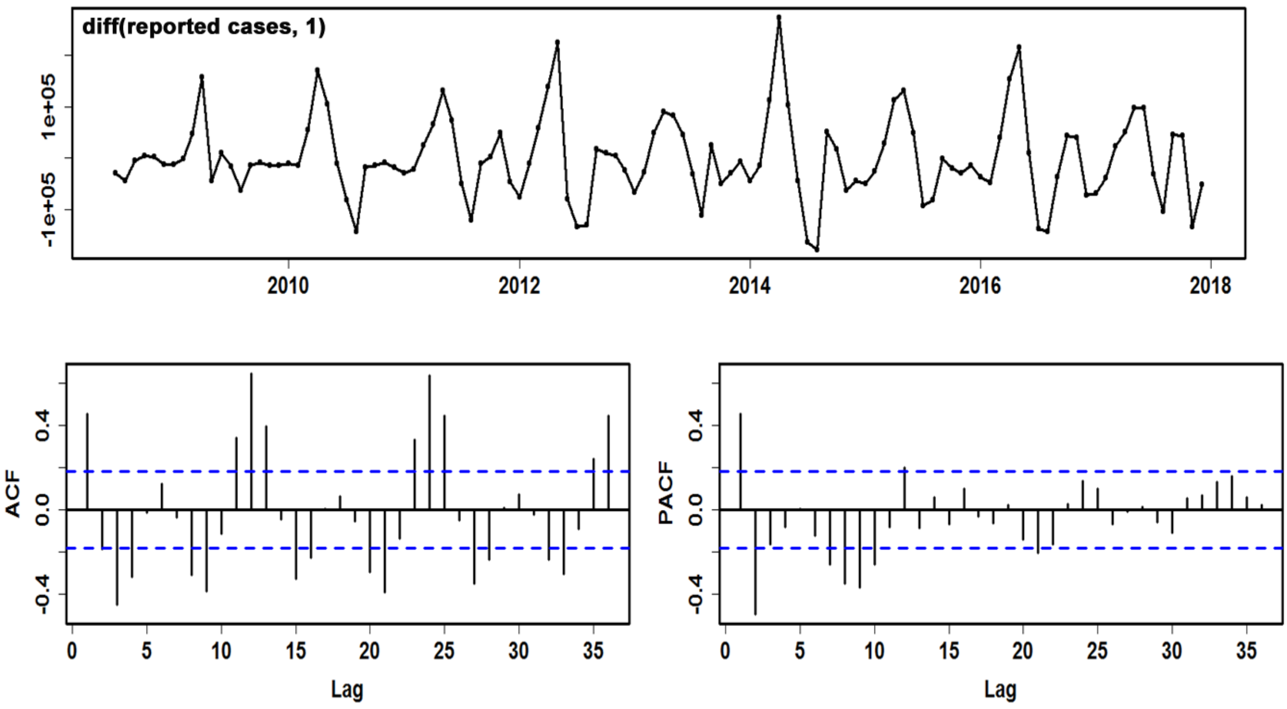

Figure S9. Autocorrelation function(ACF) and partial autocorrelation function(PACF) graphs the first-order non-seasonal difference(d=1) of monthly HFMD incidence series in mainland China from June 2008 to December 2017. The plot shows that the differenced series looks much more stationary when compared with the original time series. Nonetheless there still is an obvious seasonal pattern in this differenced series.

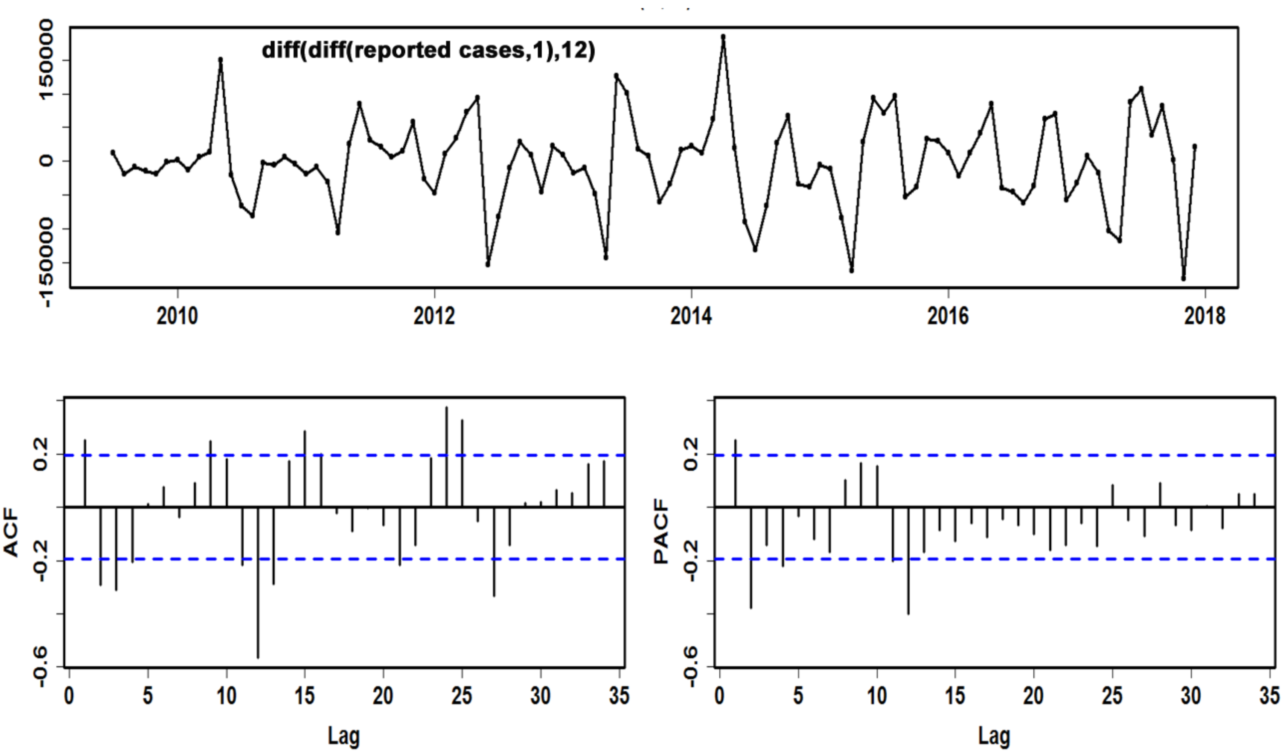

Figure S10. Autocorrelation function(ACF) and partial autocorrelation function(PACF) graphs with the first-order seasonal difference(D=1) of monthly HFMD incidence series in mainland China from June 2008 to December 2017. On the basis of the plot, we can observe that this differenced series meets the need of modeling for SARIMA method.

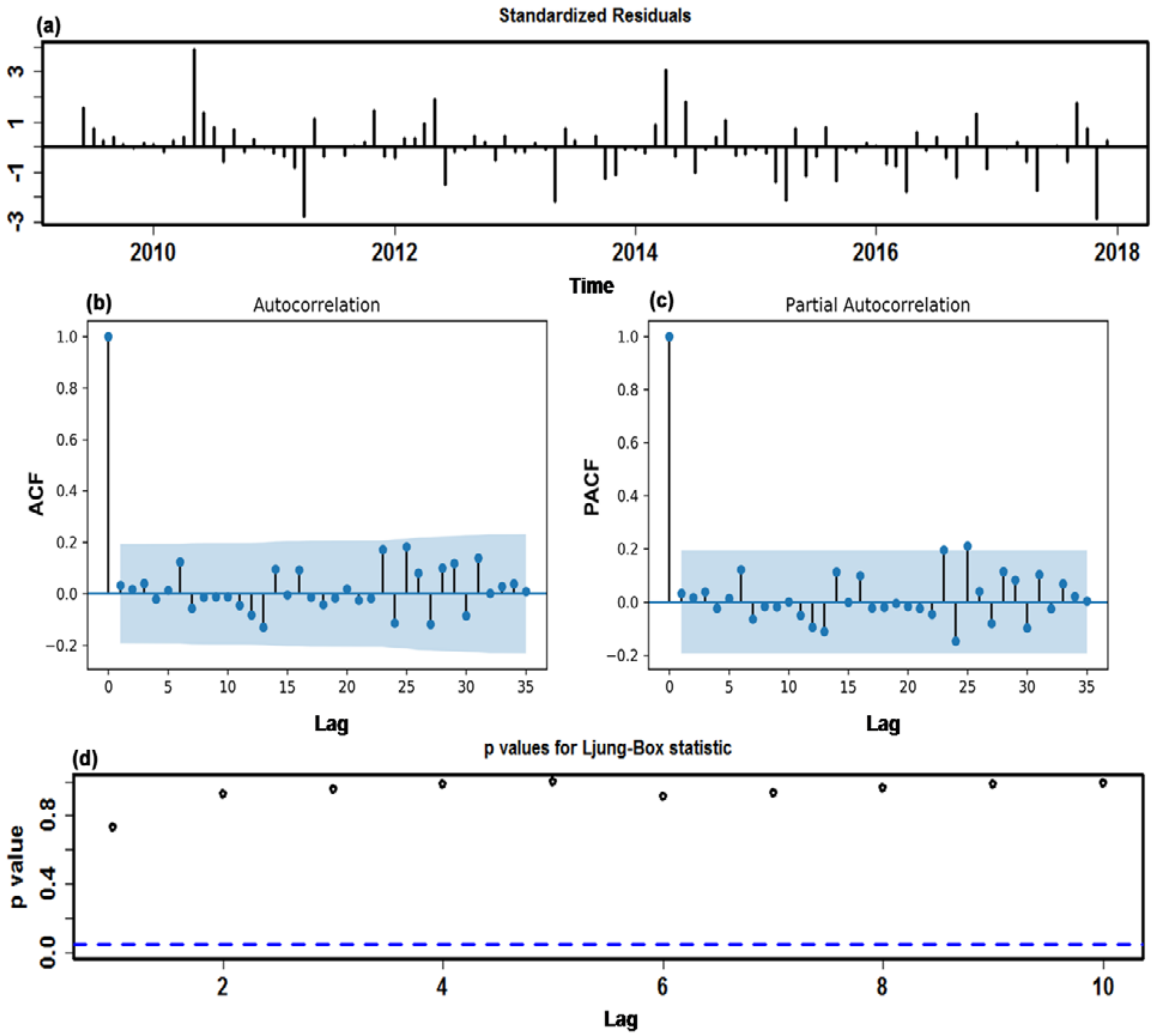

Figure S11. The resultant plots of fit goodness tests from SARIMA(1,0,1)(1,1,1)$_{12}$ model for HFMD notified cases series from June 2008 to December 2017. (a) Standardized residuals. (b) Autocorrelation function (ACF) graph of errors across varying lag times. The spikes all fall within the 95% confidence intervals in this residual series. (c) Partial autocorrelation function (PACF) graph of errors. (d) *P* values for Ljung-Box statistic.. There are large *P* values at the significance level of 5%. Diagnostic checking indicates the chosen SARIMA specification is suitable for the HFMD notified cases series from June 2008 to December 2017.

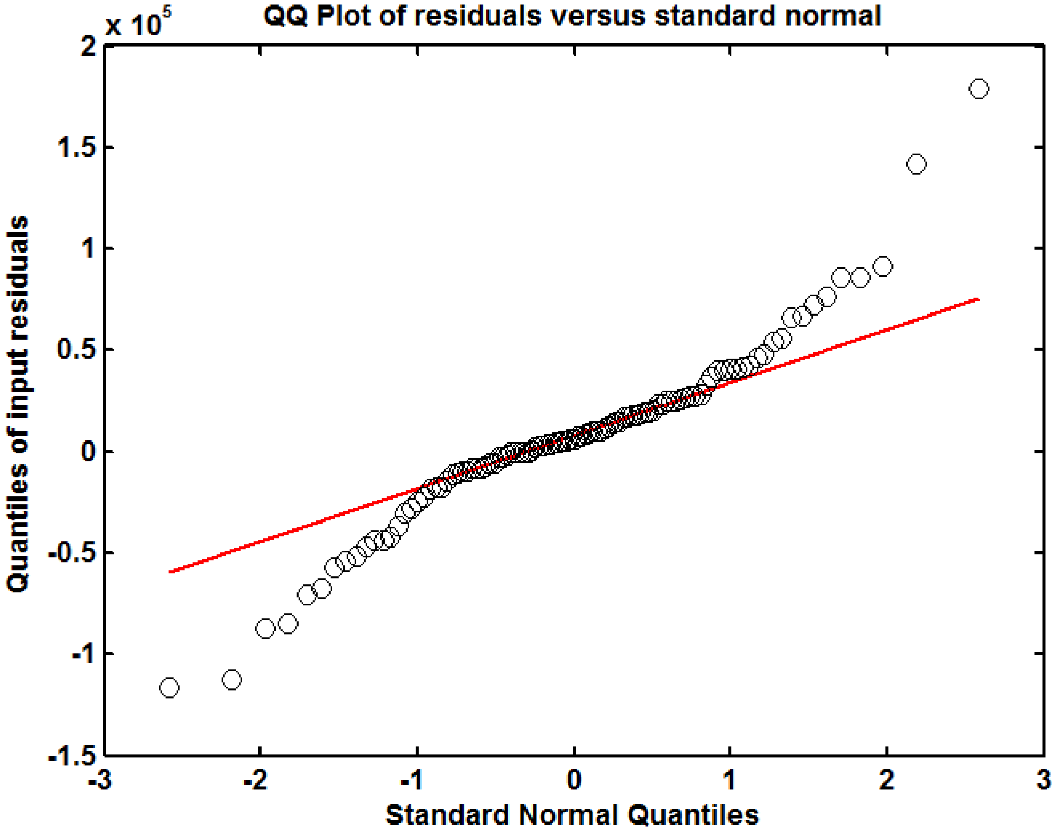

Figure S12. The Q-Q plot of residuals from SARIMA(1,0,1)(1,1,1)$_{12}$ model for HFMD series from June 2008 to December 2017. This plot suggests that residuals are departure from normality at the tails.

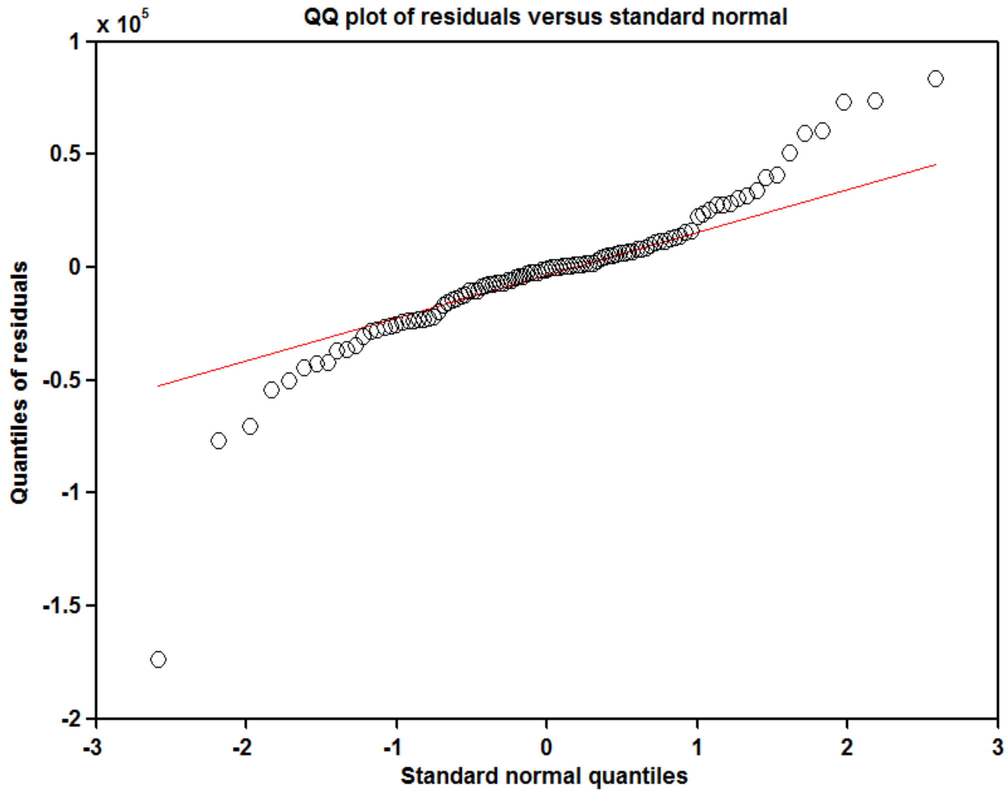

Figure S13. The Q-Q plot of residuals from NAR model for HFMD series from June 2008 to June 2017. The Q-Q plot of the residuals shows departure from normality at the tails.

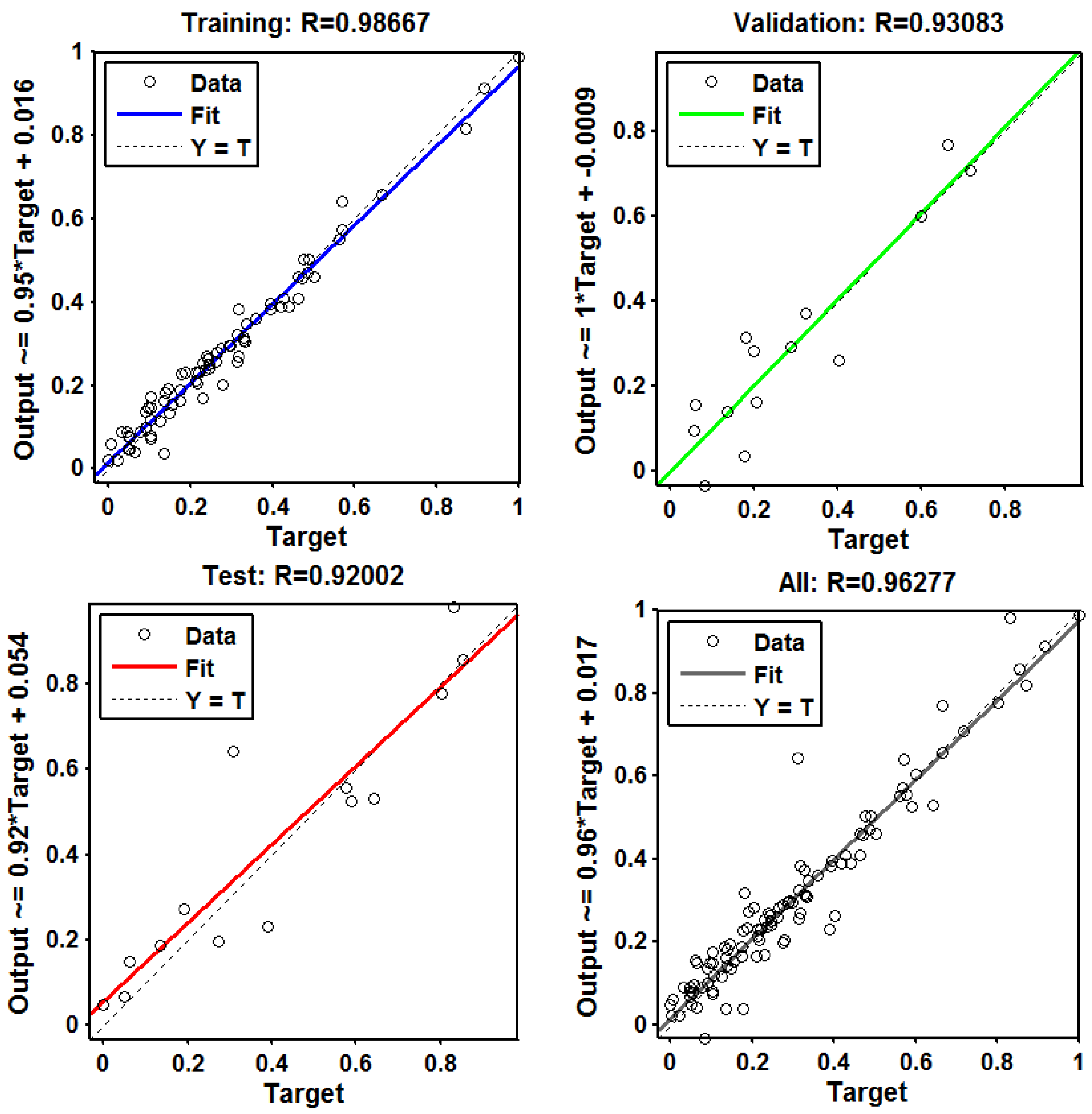

Figure S14. The regression plots for the NAR model outputs with respect to targets for training, validation, and test in the dataset from June 2008 to June 2017.

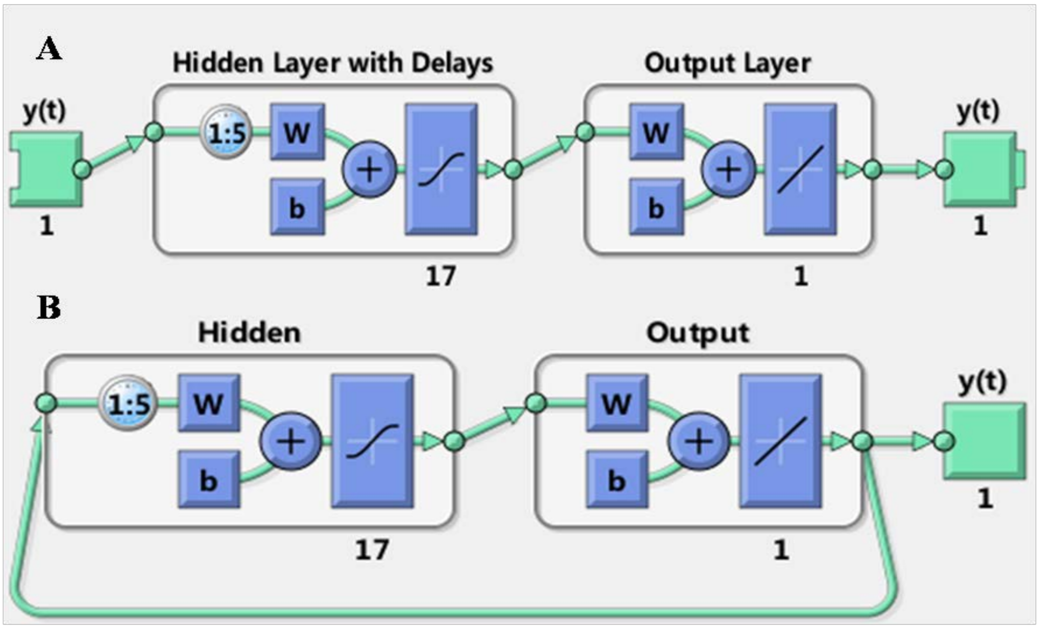

Figure S15. The layer architecture of NAR model for the HFMD notified cases series from June 2008 to December 2016. (A) The opened loop mode; (B) The closed loop mode. This NAR model is comprised of a hidden layer with 17 neurons and 5 delays and an output layer with 1 neuron. The model adopts tapped delay lines to store prior data of the x(t) and y(t) series as well. Among which, the output results of the model, y(t), is fed back to the input (through delays), since y(t) is a function of y(t – 1), y(t – 2), ...,y(t – d). Nevertheless, in order to train more efficiently, the training can be undertaken in open loop. After training, then the opened loop mode should be transformed to the closed loop mode for multistep-ahead forecasting.

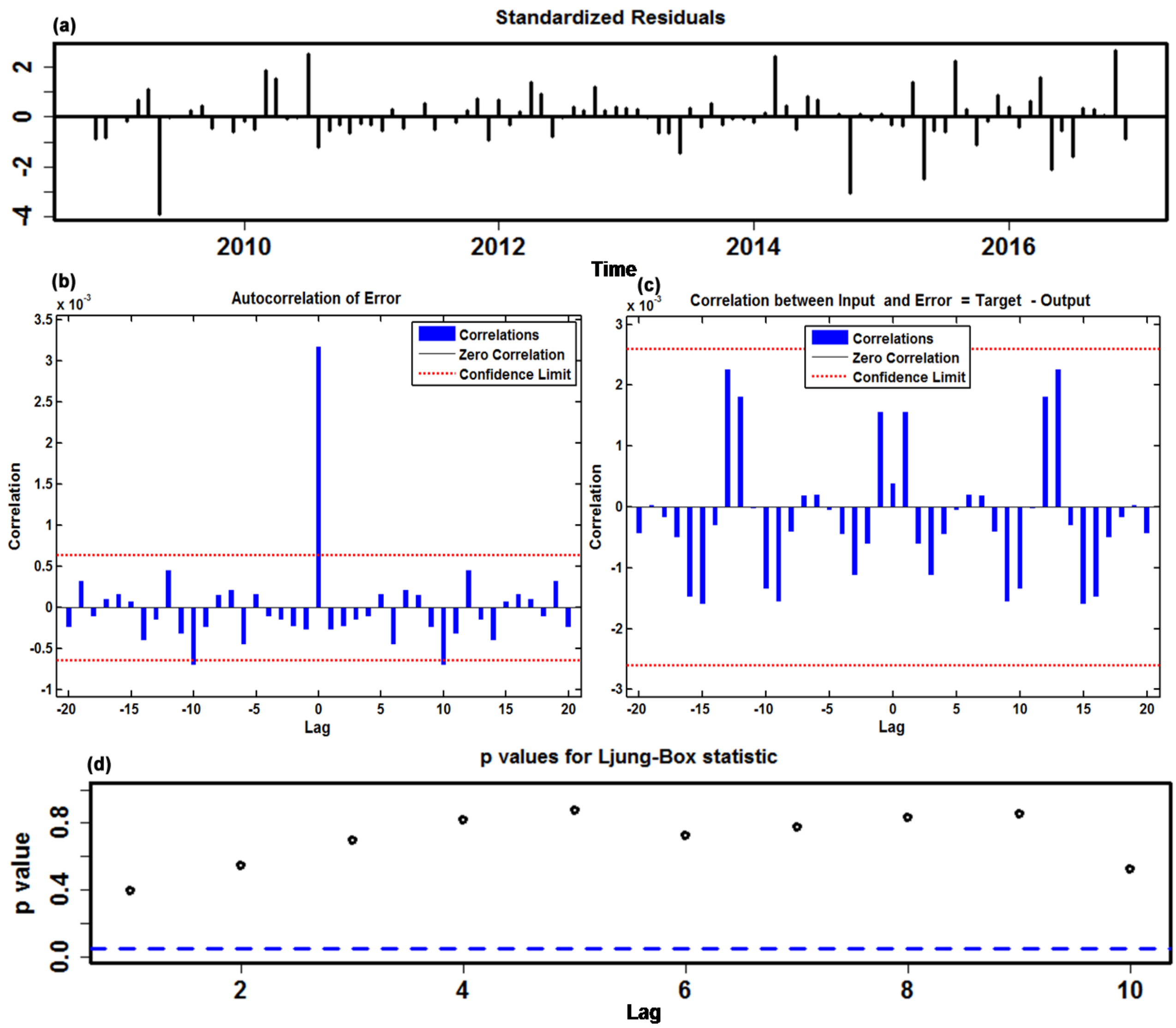

Figure S16. The resulting plots of fit goodness tests from the best-fitting NAR model for HFMD notified cases series from June 2008 to December 2016. (**a**) Standardized residuals. (**b**) Autocorrelation function (ACF) plot of errors across varying lag times. All of the autocorrelations fail to be beyond the estimated 95% uncertainty bounds around zero across varying lag times apart from the one from ACF plot at zero lag that should occur and also occurring at lag 11, we should not be surprised as this can easily happen by chance alone. Overall, the plot manifests that the network appears to have captured the dependence hidden behind the HFMD notified cases series. (**c**) Input-to-error correlation plot for varying lags. The input-error cross-correlation function illustrates how the residuals are interrelated with the series of x(t). All of the correlations fall within the confidence bounds around zero, which hints the developed model is a perfect specification. (**d**) Q-statistic *P*-values. Analyses form the plots demonstrate the constructed model is adequate in excavating the information of this time series.

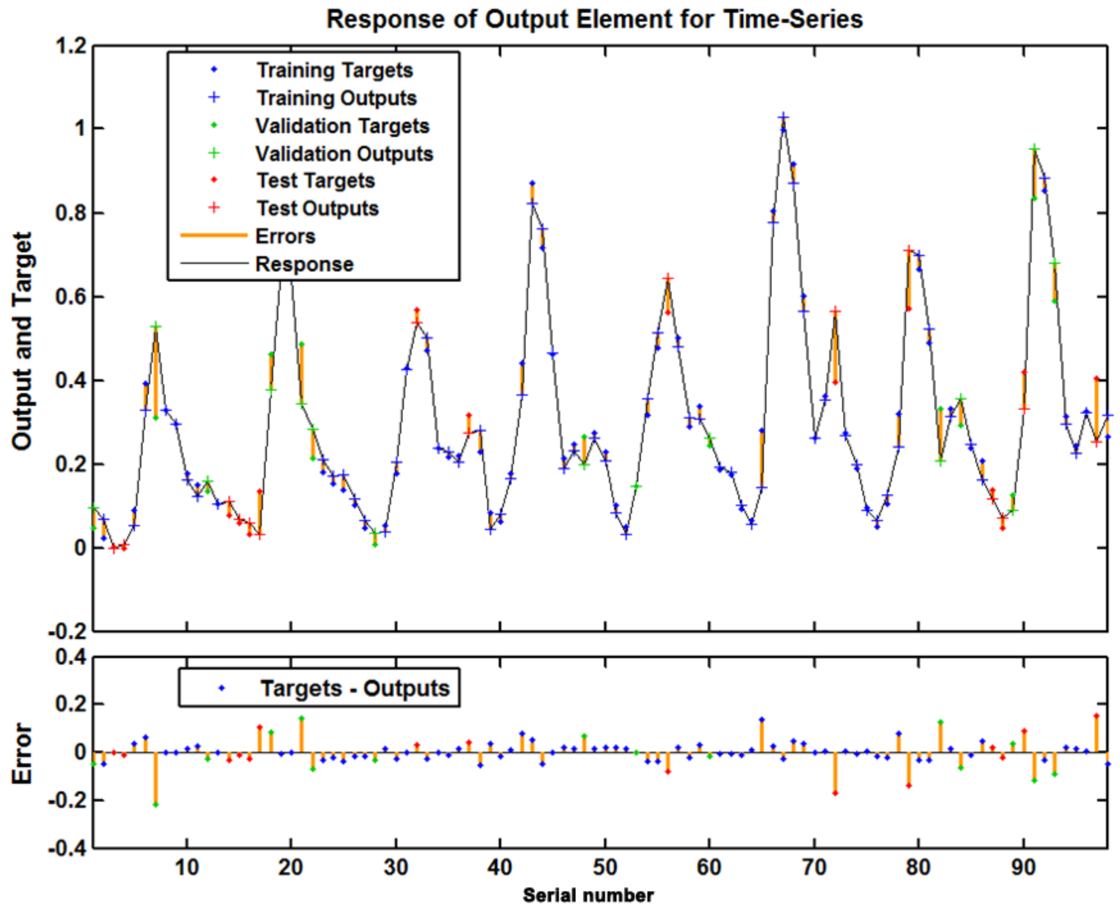

Figure S17. The response of output and target for HFMD time series from June 2008 to December 2016 at various time points. This plot exhibits which time points are elected as the training, validation and testing subsets, along with their corresponding errors between inputs and targets. In view of the small errors, a further suggestion that the fitting is fairly accurate.

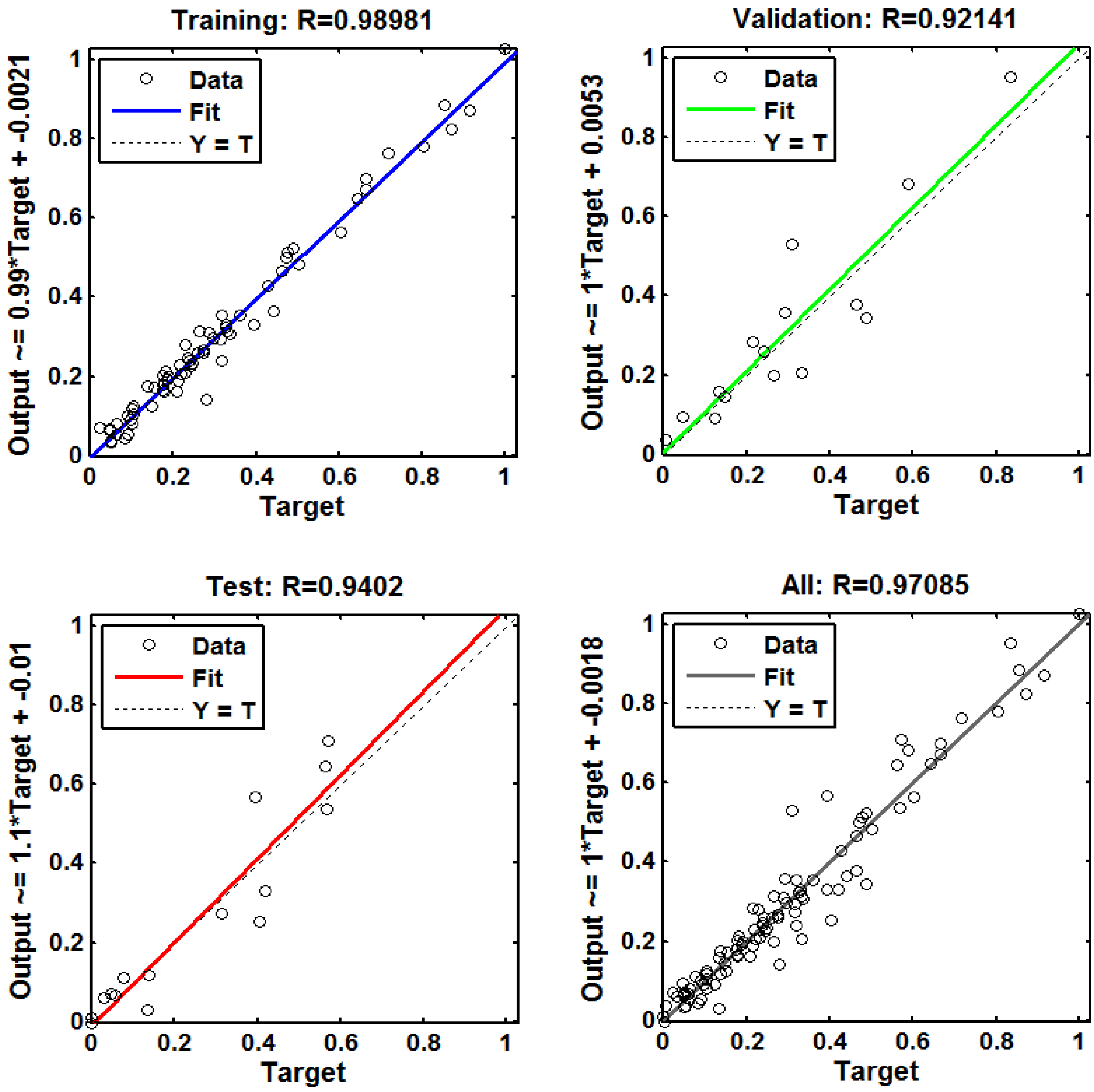

Figure S18. The regression plots for the best-fitting NAR model outputs with respect to targets for training, validation, and test in the dataset from June 2008 to December 2016.

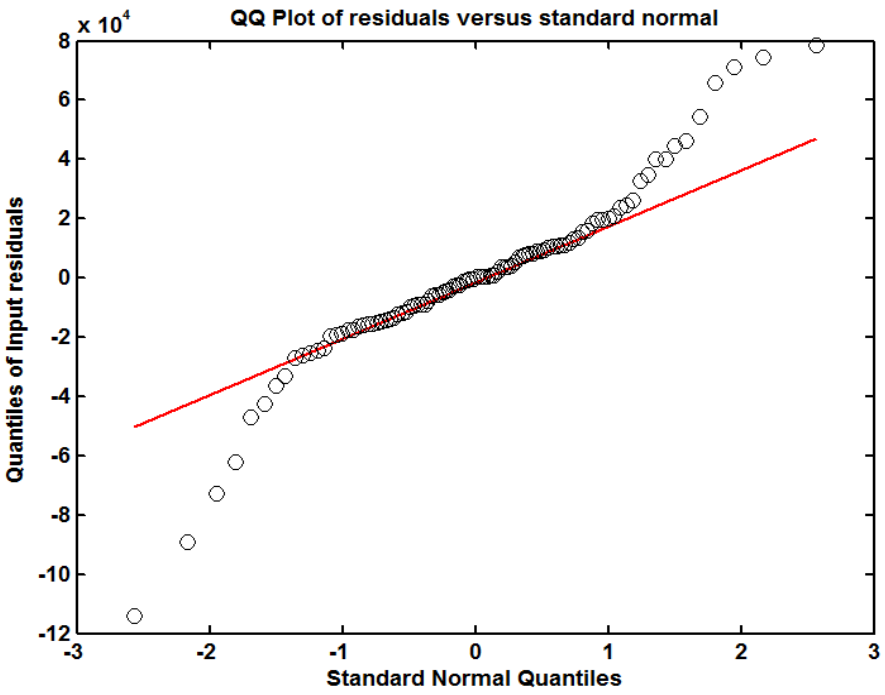

Figure S19. The Q-Q plot of residuals from NAR model for HFMD series from June 2008 to December 2016. The Q-Q plot of the residuals shows marked departure from normality at the tails.

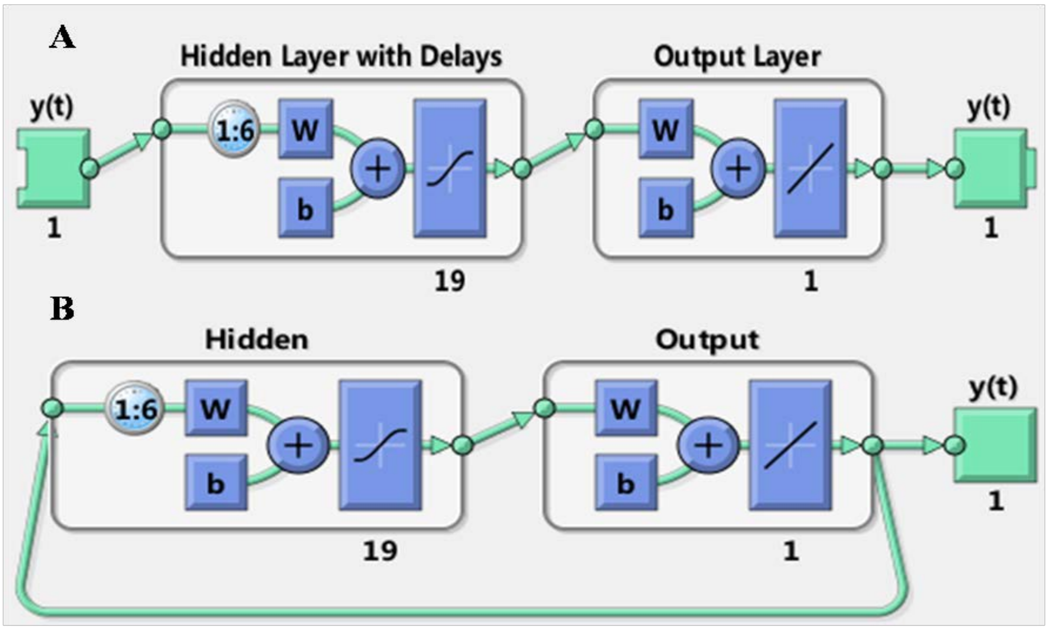

Figure S20. The layer architecture of NAR model for HFMD notified cases series from June 2008 to December 2017. (A) The opened loop mode; (B) The closed loop mode. This NAR model is comprised of a hidden layer with 19 neurons and 6 delays and an output layer with 1 neuron. The model adopts tapped delay lines to store prior data of the x(t) and y(t) series as well. Among which, the output results of the model, y(t), is fed back to the input (through delays), since y(t) is a function of y(t – 1), y(t – 2), ...,y(t – d). Nevertheless, in order to train more efficiently, the training can be undertaken in open loop. After training, then the opened loop mode should be transformed to the closed loop mode for multistep-ahead forecasting.

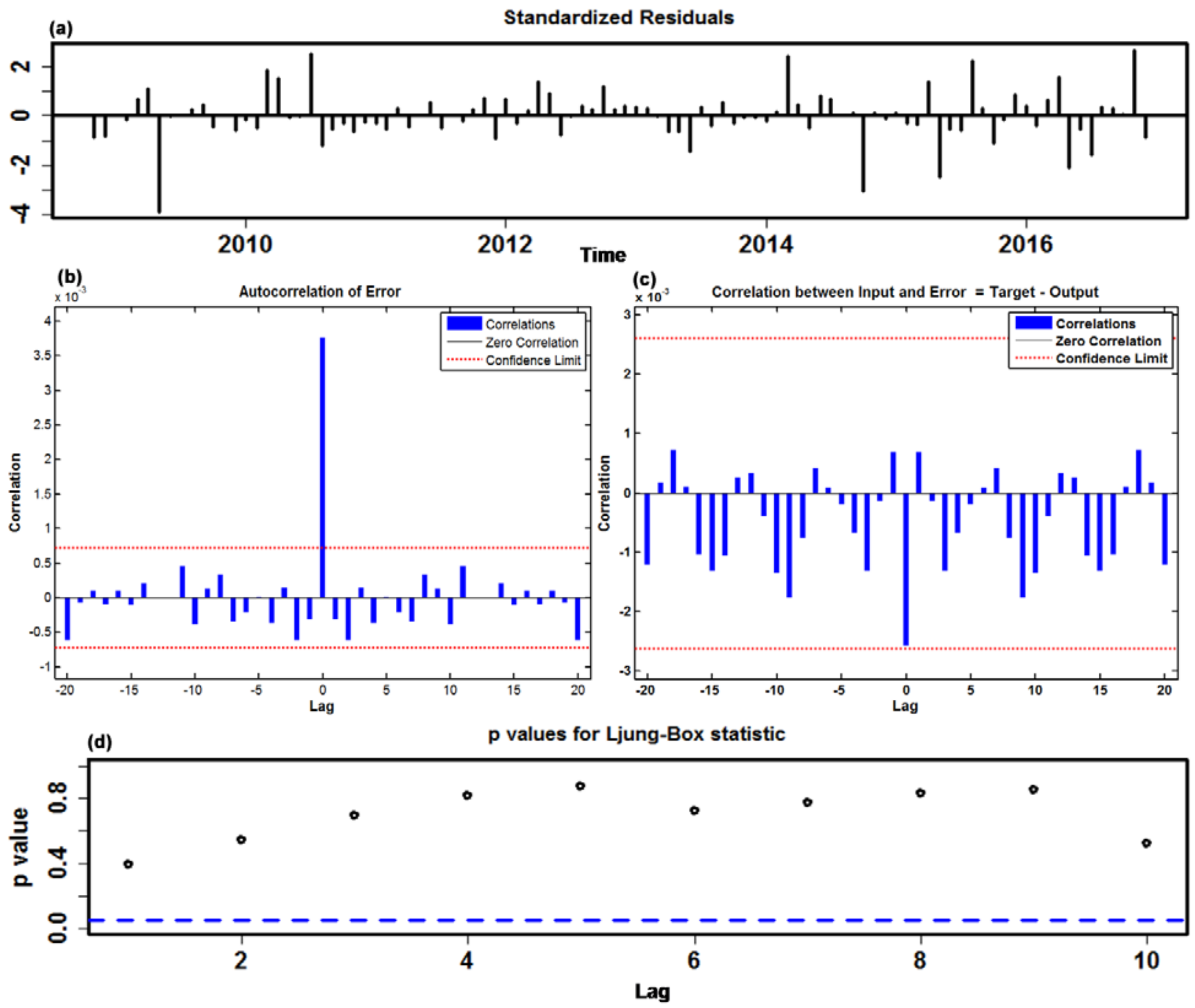

Figure 21. The resulting plots of fit goodness tests from the best-fitting NAR model for HFMD notified cases series from June 2008 to December 2017. (**a**) Standardized residuals. (**b**) Autocorrelation function (ACF) plot of errors across varying lag times. All of the autocorrelations fail to be beyond the estimated 95% uncertainty bounds around zero across varying lag times apart from the one from ACF plot at zero lag that should occur. The plot manifests that the network appears to have captured the dependence hidden behind the HFMD notified cases series. (**c**) Input-to-error correlation plot for varying lags. The input-error cross-correlation function illustrates how the residuals are interrelated with the series of x(t). All of the correlations fall within the confidence bounds around zero, which hints the developed model is a perfect specification. (**d**) Q-statistic *P*-values. Analyses form the plots demonstrate the constructed model is adequate in excavating the information of this time series.

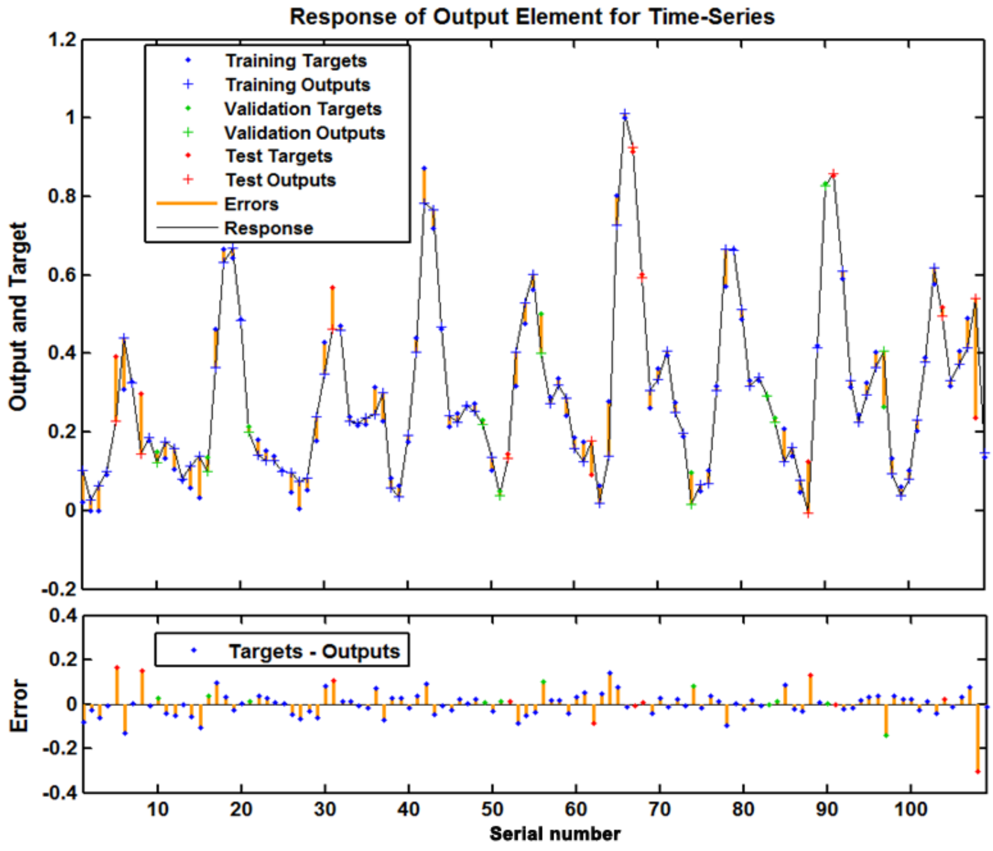

Figure S22. The response of output and target for HFMD time series from June 2008 to December 2017 at various time points. This plot exhibits which time points are elected as the training, validation and testing subsets, along with their corresponding errors between inputs and targets. In view of the small errors nearly lying between -0.2 and 0.2, a further suggestion that the fitting is fairly accurate.

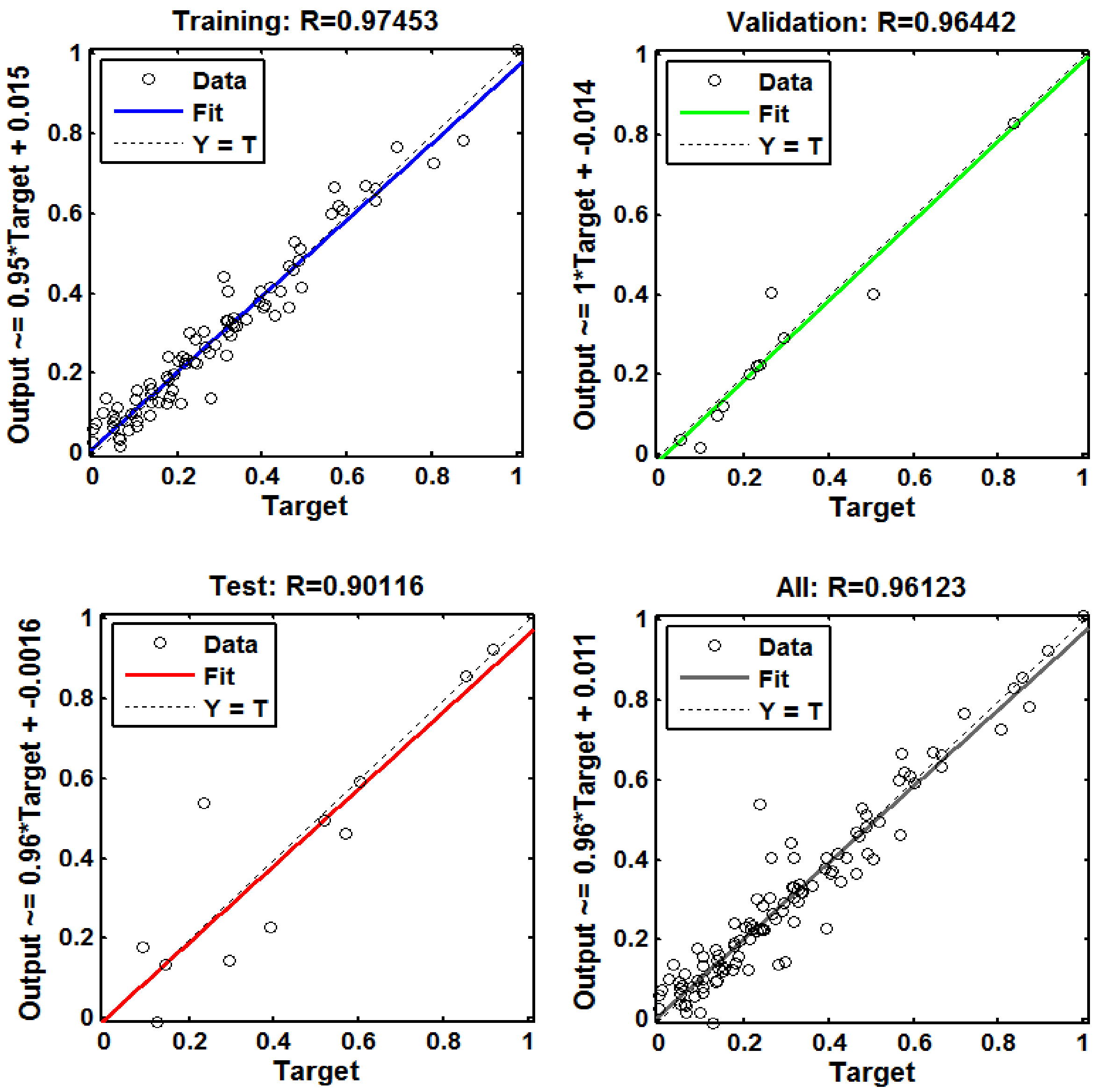

Figure S23. The regression plots for the best-fitting NAR model outputs with respect to targets for training, validation, and test in the dataset from June 2008 to December 2017.

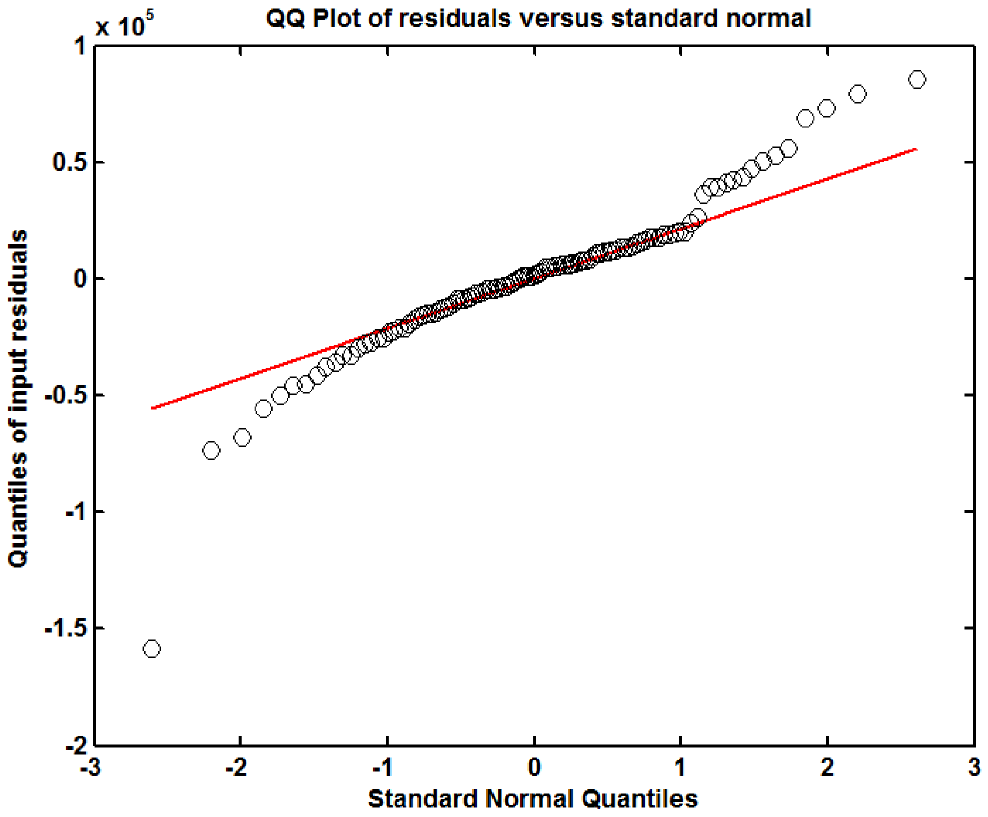

Figure S24. The Q-Q plot of residuals from the best-simulating NAR model for HFMD series from June 2008 to December 2017. This plot suggests that the distribution of produced residuals has a tail thicker than that of a normal distribution.

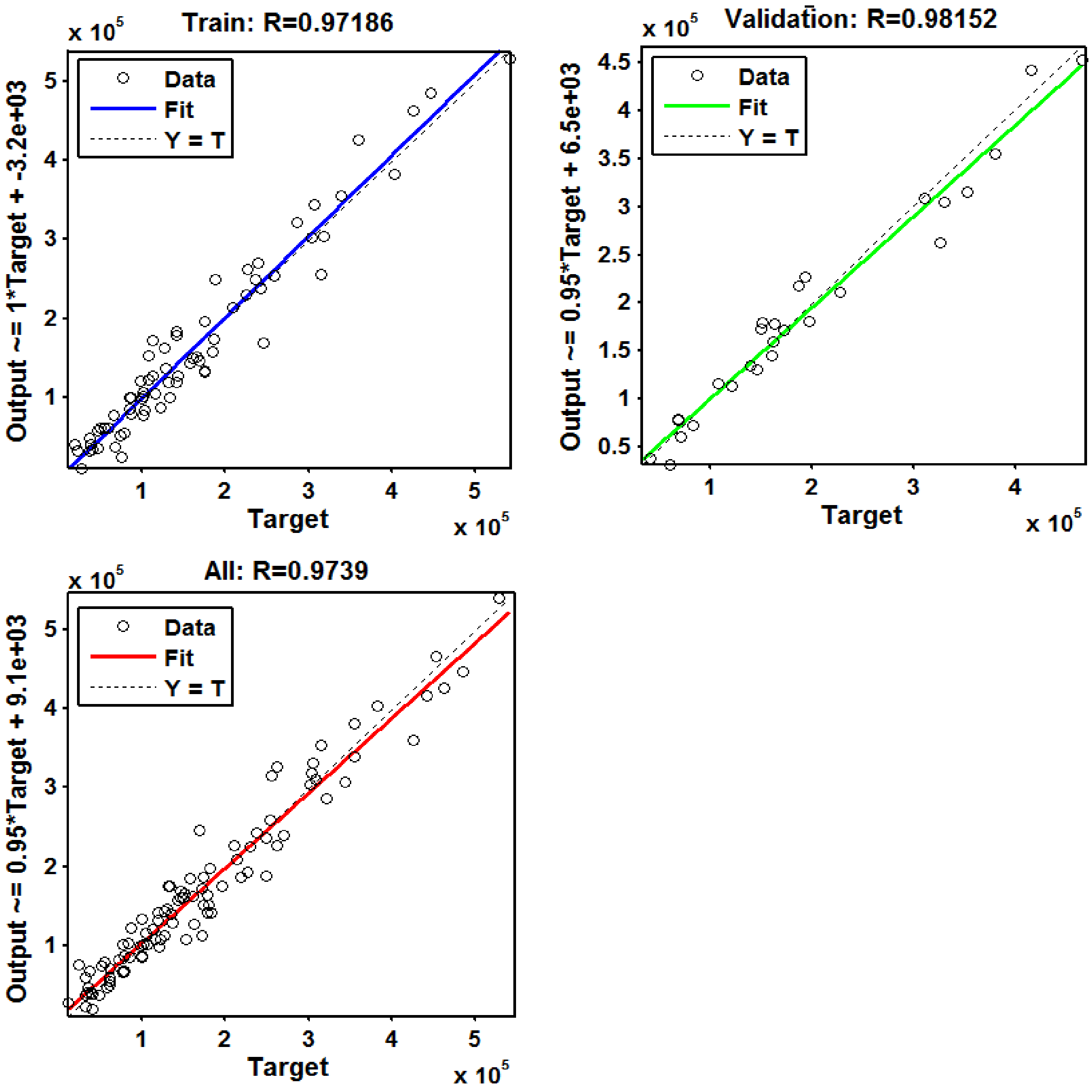

Figure S25. The regression plots for the LSTM model outputs with respect to targets for training and validation in the dataset from June 2008 to June 2017.

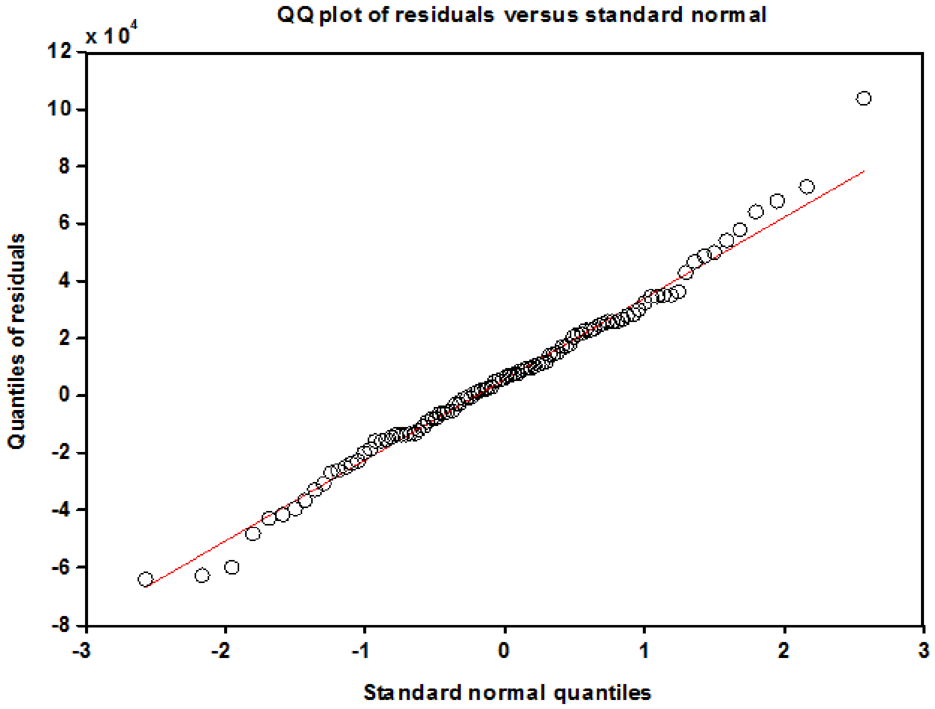

Figure S26. The Q-Q plot of residuals from LSTM model for HFMD series from June 2008 to June 2017. As exhibited in the plot of the normal Q-Q plot, the residuals approximately fall along the line. Thus the best-fitting LSTM model improves the normality dramatically compared with the basic NAR and SARIMA methods.

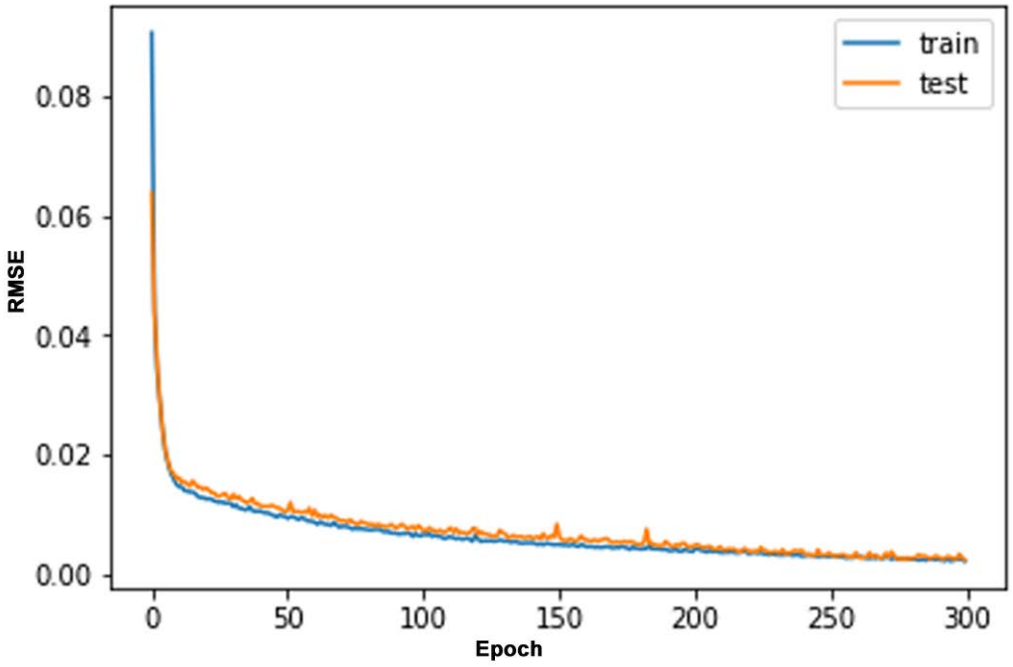

Figure S27. The training and validation performances for LSTM model at 300 epochs for the HFMD notified cases series from June 2008 to December 2016. This plot documents that the test set error and the validation set error have similar characteristics and no significant overfitting has occurred by iteration 300.

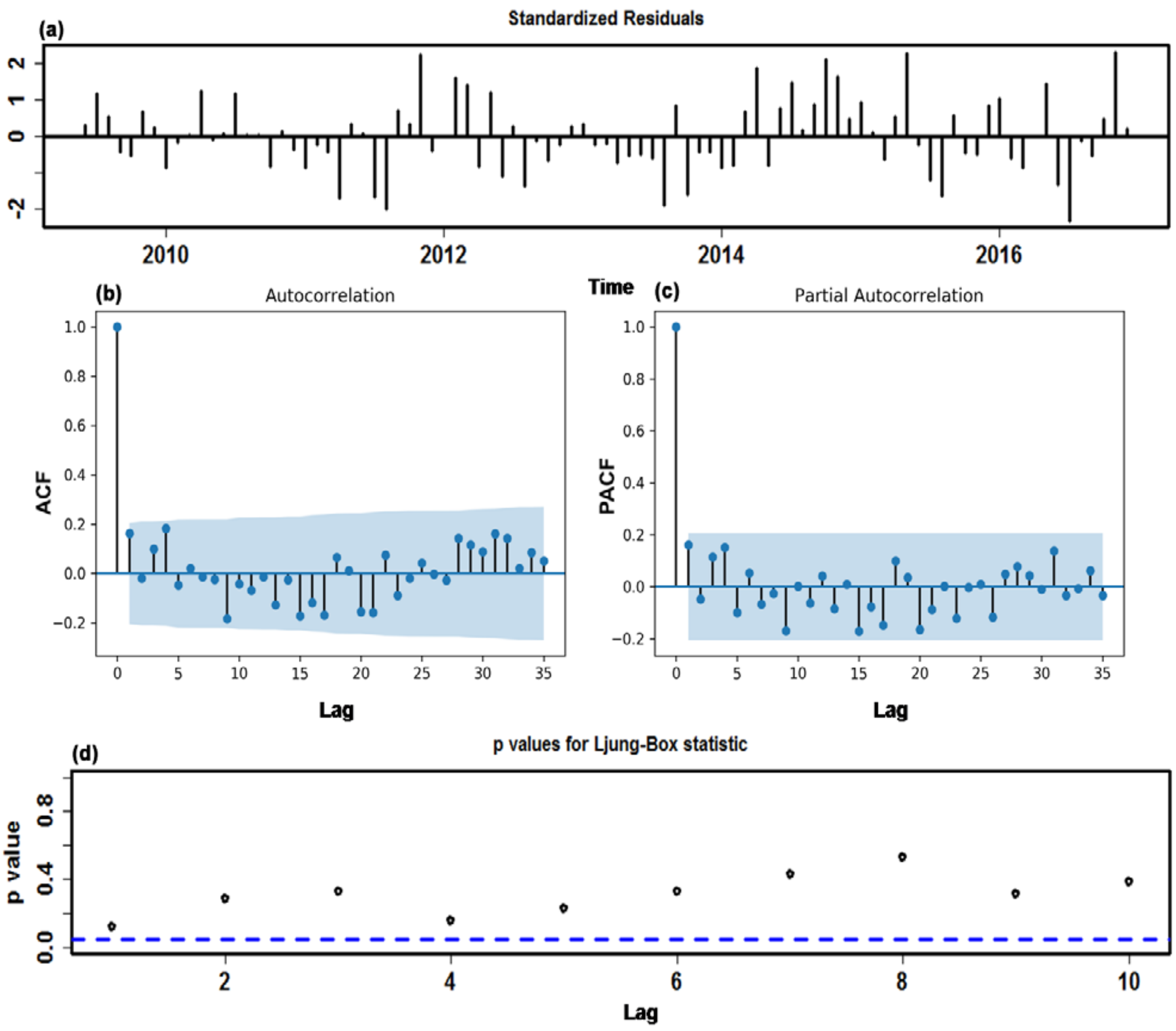

Figure S28. The resulting plots of fit goodness tests from the best-performing LSTM model for HFMD notified cases series from June 2008 to December 2016. (**a**) Standardized residuals. (**b**) Autocorrelation function (ACF) plot of errors across varying lag times. The ACF plot of forecasted errors reveals no individually evident autocorrelation at varying lags except for the one from ACF plot at zero lag that should occur. (**c**) Partial autocorrelation function (PACF) plot of residuals. (**d**) Q-statistic *P*-values. As shown, All *P*-values are larger than 0.05. These diagnostics manifest that the network is well suited to the dataset.

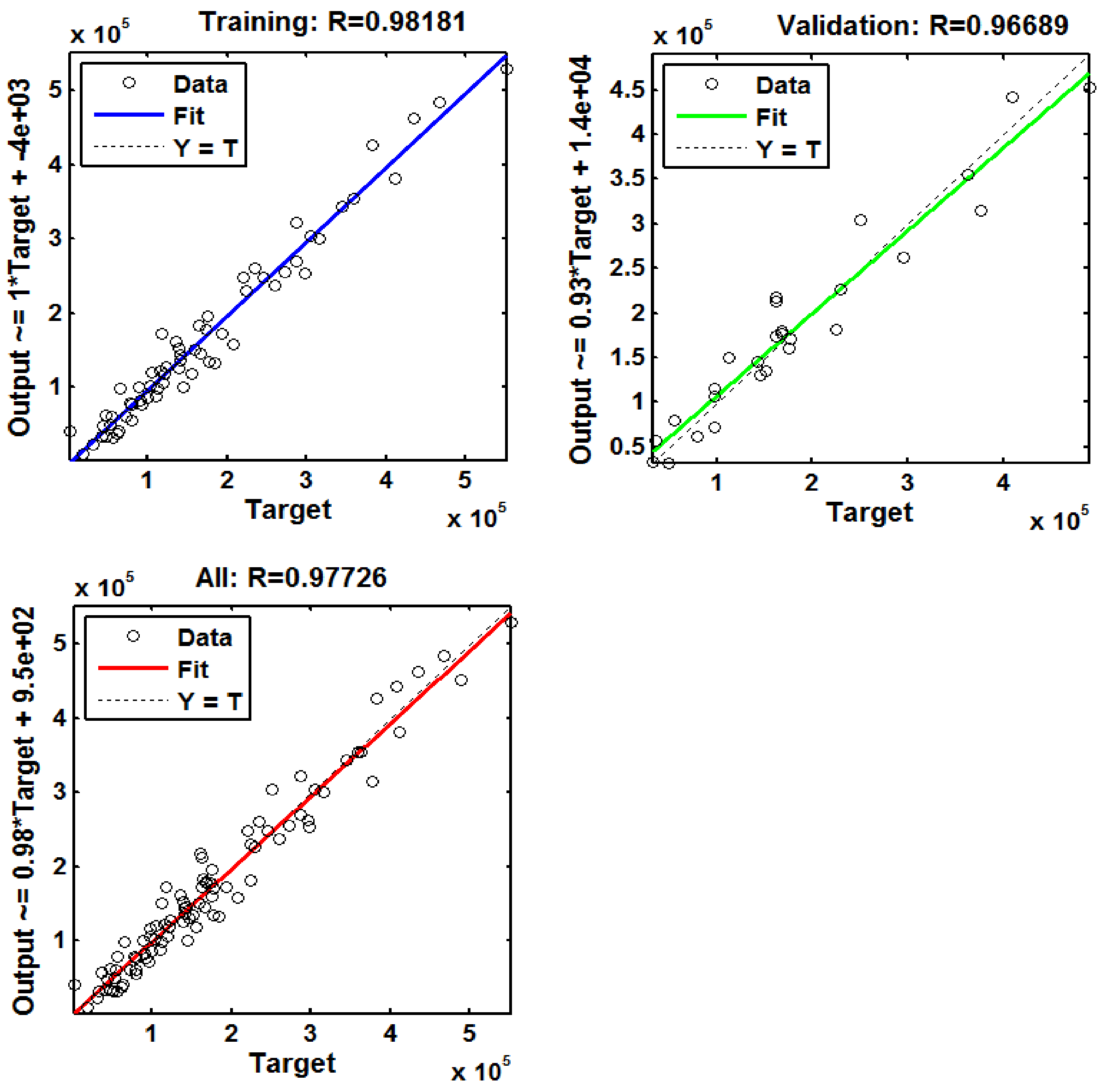

Figure S29. The regression plots for the best-presenting LSTM model outputs with respect to targets for training and validation in the dataset from June 2008 to December 2016.

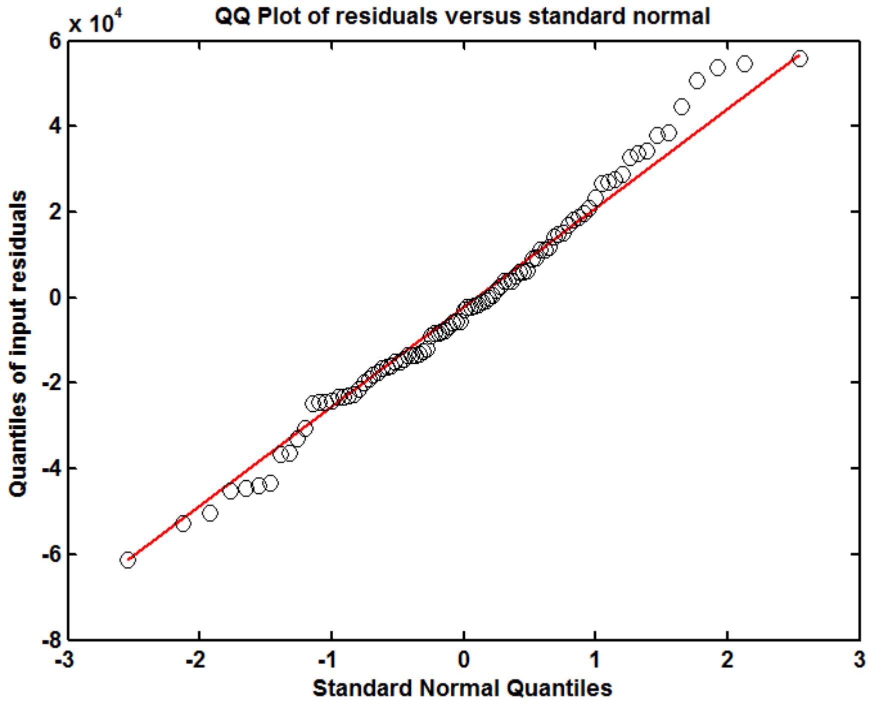

Figure S30. The Q-Q plot of residuals from LSTM model for HFMD series from June 2008 to December 2016. As exhibited in the plot of the normal Q-Q plot, the points seem to follow the straight line fairly closely. This graph would not lead us to reject normality of the error terms in this model. Thus the best-fitting LSTM model improves the normality dramatically compared with the basic NAR and SARIMA methods.

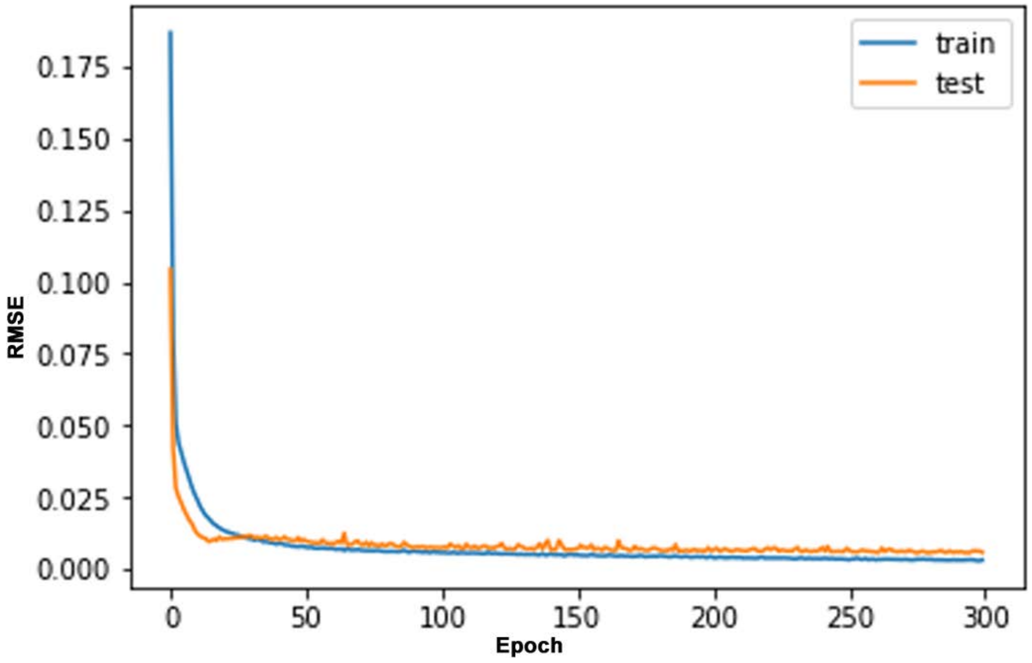

Figure S31. The training and validation performances for LSTM model at 300 epochs for the HFMD notified cases series from June 2008 to December 2017. This plot documents that the test set error and the validation set error have similar characteristics and no significant overfitting has occurred by iteration 300.

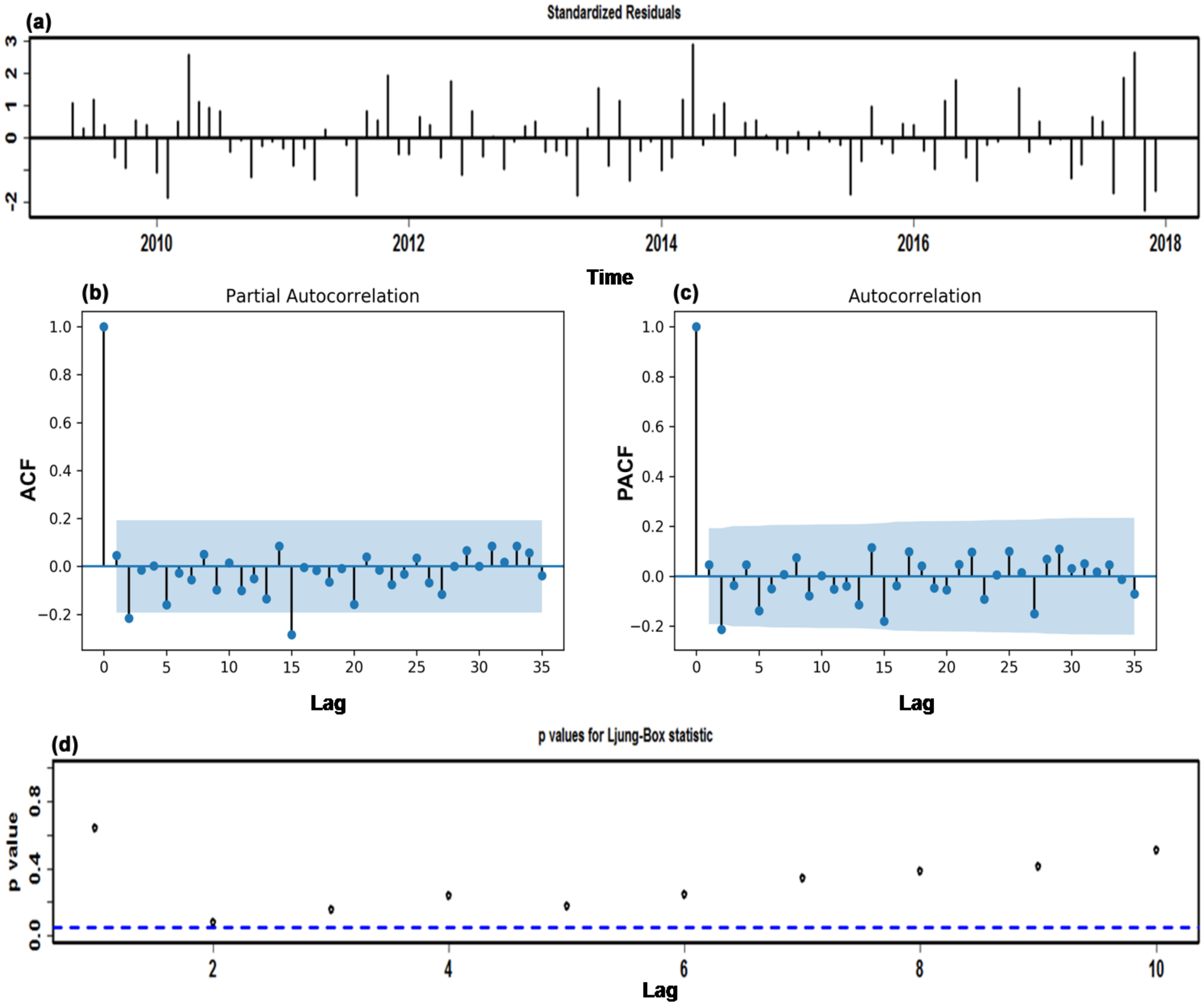

Figure S32. The resulting plots of fit goodness tests from the LSTM model for HFMD notified cases series from June 2008 to December 2017. (**a**) Standardized residuals. (**b**) Autocorrelation function (ACF) plot of errors across varying lag times. The ACF plot of forecasted errors reveals no individually evident autocorrelation at varying lags except for the two points occurring at lags 2 and 15. For these two lagged points out of the estimated 95% confidence limit, they are also reasonable as this phenomenon can easily happen by chance alone. (**c**) Partial autocorrelation function (PACF) plot of residuals. (**d**) Q-statistic *P*-values. As shown, All *P*-values are larger than 0.05. These diagnostics manifest that the network is well suited to the dataset.

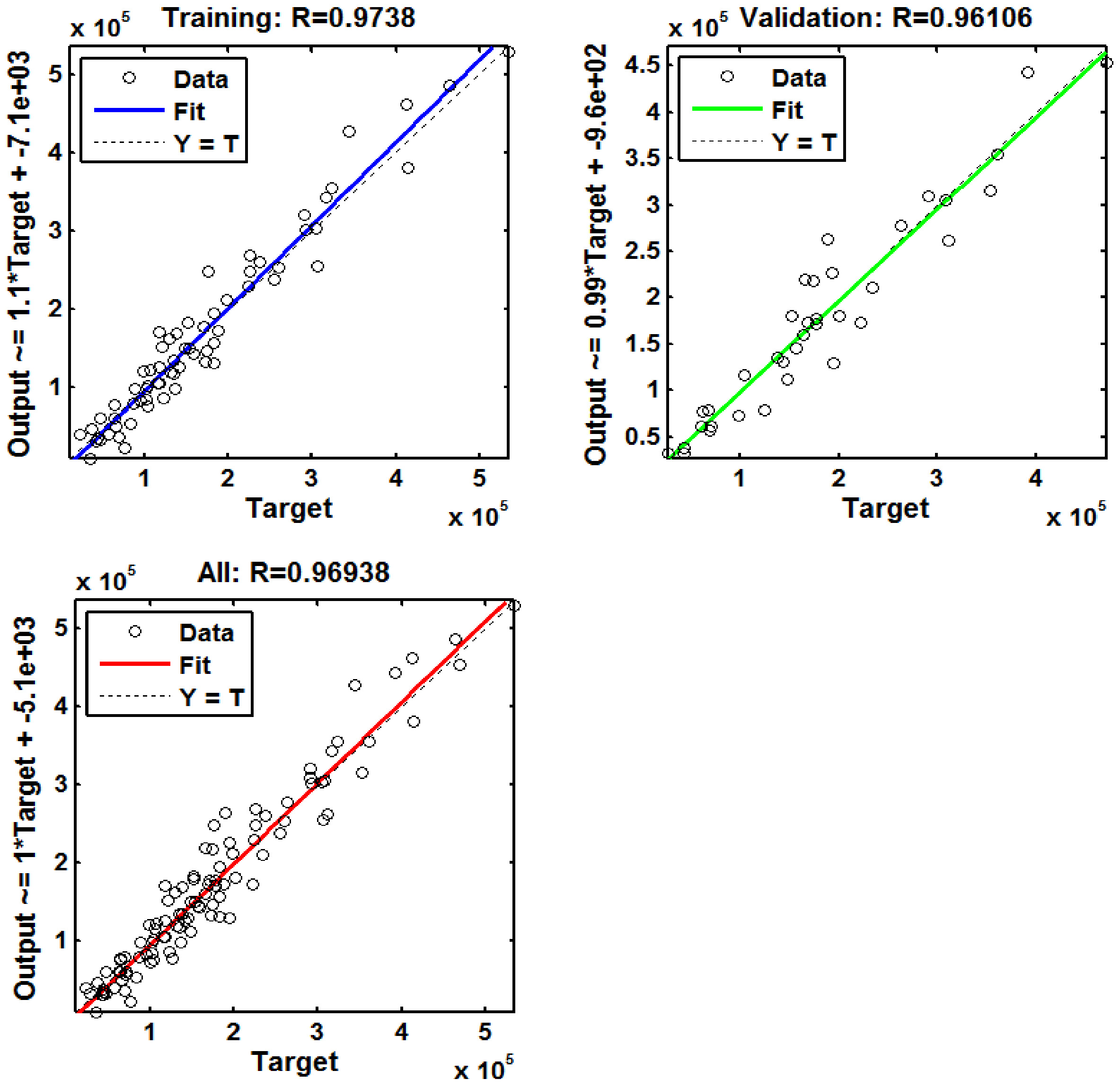

Figure S33. The regression plots for the best-presenting LSTM model outputs with respect to targets for training and validation in the dataset from June 2008 to December 2017.

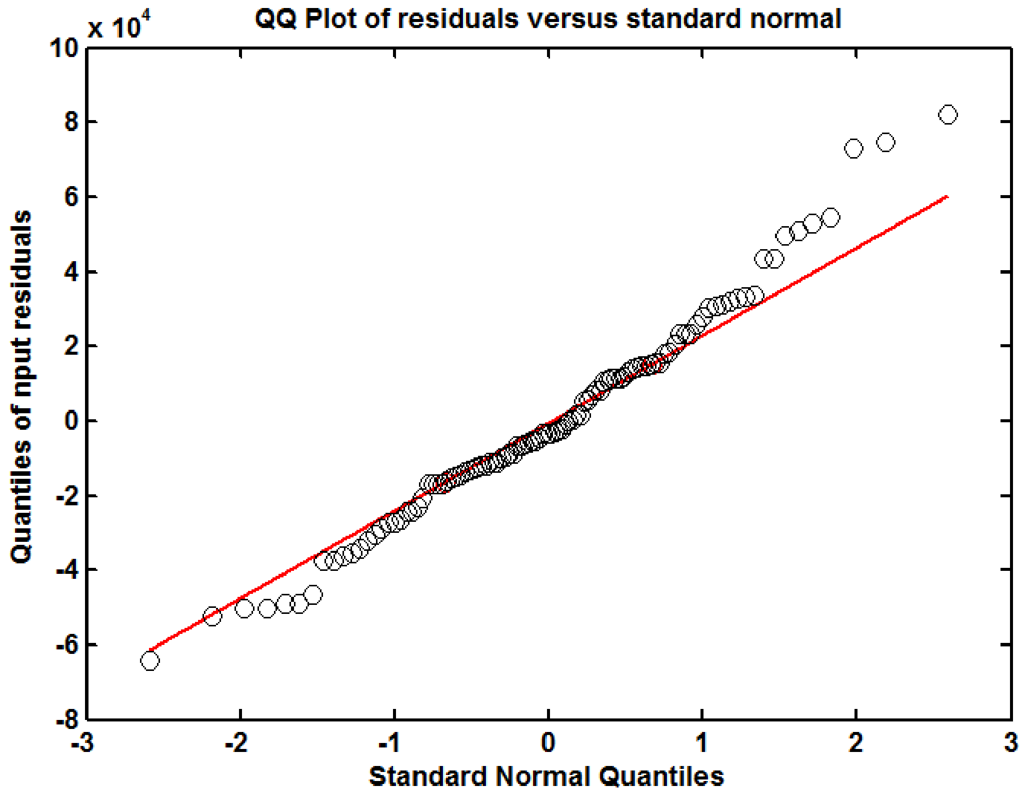

Figure S34. The Q-Q plot of residuals from LSTM model for HFMD series from June 2008 to December 2017. The Q-Q plot suggests that the distribution of errors may have a tail thicker than that of a normal distribution and may be somewhat skewed to the right. However, In comparison with the best-mimicking NAR and SARIMA approaches, the best-fitting LSTM model can improve the normality dramatically.

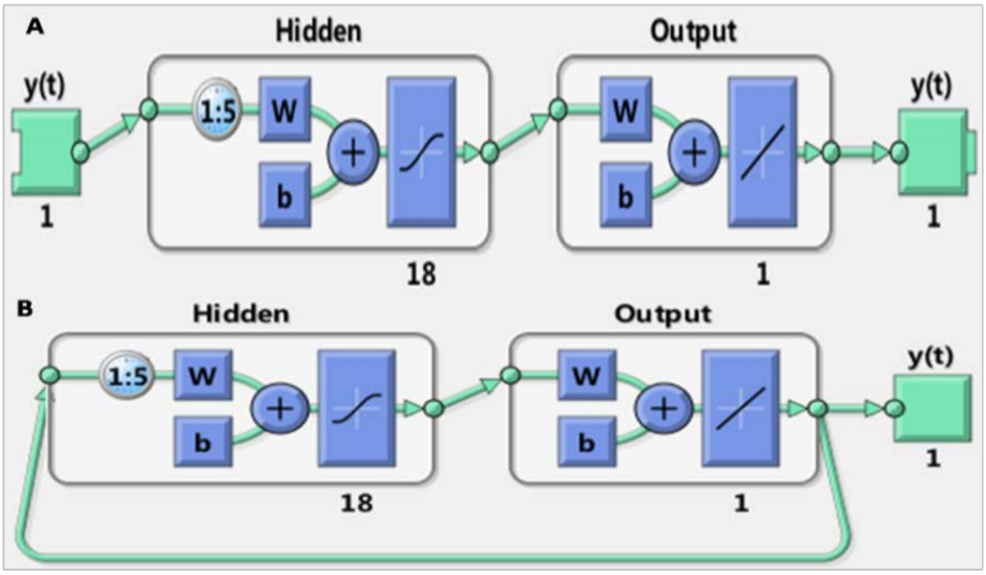

Figure S35. The layer architecture of NAR model for the HFMD notified cases series from June 2008 to June 2017. (A) The opened loop mode; (B) The closed loop mode. This NAR model is comprised of a hidden layer with 18 neurons and 5 delays and an output layer with 1 neuron. The model adopts tapped delay lines to store prior data of the x(t) and y(t) series as well. Among which, the output results of the model, y(t), is fed back to the input (through delays), since y(t) is a function of y(t – 1), y(t – 2), ...,y(t – d). Nevertheless, in order to train more efficiently, the training can be undertaken in open loop. After training, then the opened loop mode should be transformed to the closed loop mode for multistep-ahead forecasting.

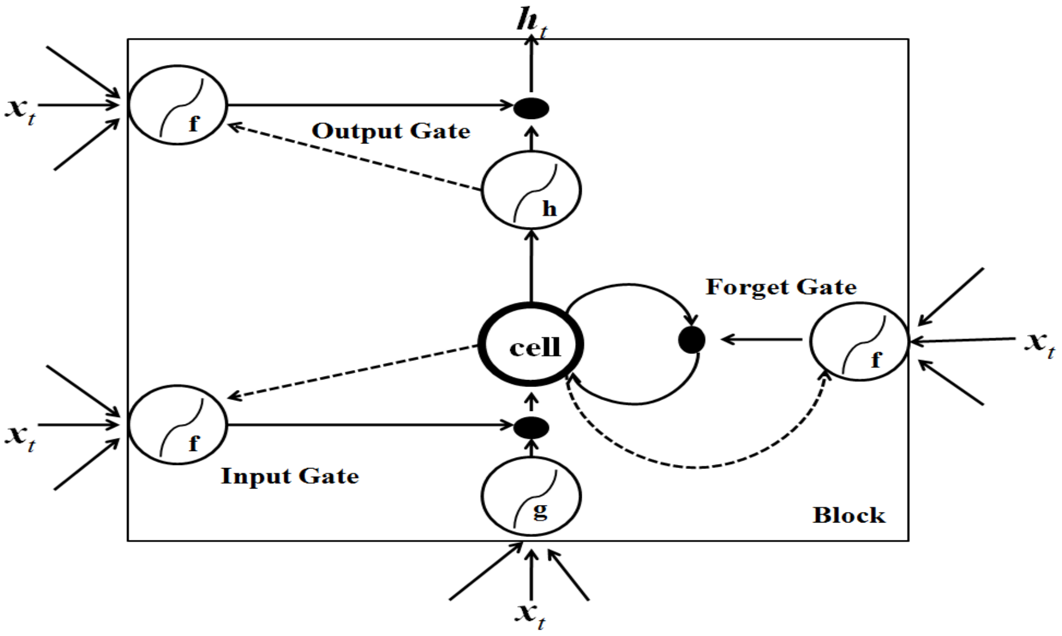

Figure S36. The layer architecture of LSTM model. Above-mentioned these gates represent nonlinear summation units that gather activations from inside and outside the block, and dominate the activation of the cell via multiplications (small black circles). The input and output gates multiply the cell's input and output when the forget gate multiplies the earlier state of the cell. The gate activation function 'f' ordinarily refers to the logistic sigmoid, which can limit the gate activations into [0, 1] intervals. The cell input and output activation functions ('g' and 'h') customarily stand for tanh or logistic sigmoid. In this layer architecture, dashed lines are the weighted join points from the cell to the gates, the remainder of lines within the block denote the unweighted join points. The only outputs from the block to the remaining the network emanate from the output gate multiplcication.

Models	Estimated parameter coefficients of candidate models							Performance indexes of candidate models		
	AR1	AR2	MA1	MA2	SAR1	SMA1	SMA2	AIC	AICc	BIC
ARIMA(1,0,1)(0,1,1)$_{12}$	0.6358		0.4441			-0.6747		2364.50	2364.94	2374.8
	0.1022		0.1055			0.1324				
ARIMA(2,1,1)(0,0,2)$_{12}$	1.0877	-0.5008	-0.964			0.6641	0.6982	2629.47	2630.3	2645.56
	0.0897	0.0895	0.0275			0.1246	0.1687			
ARIMA(2,1,1)(1,1,0)$_{12}$	0.8218	-0.2785	-0.9332		-0.6009			2340.16	2340.83	2352.98
	0.1158	0.1079	0.0498		0.0869					
ARIMA(1,1,2)(1,1,0)$_{12}$	0.4344		-0.5235	-0.3938	-0.6001			2339.41	2340.08	2352.23
	0.1477		0.141	0.1221	0.0874					

Table S1. The estimated parameters and performance indexes for the selected candidate models based on the original observations from June 2008 to June 2017.

Parameters	Coefficient	Standard error	*t*	*P*
AR1	0.484	0.121	3.992	<0.001
MA1	-0.432	0.133	-3.256	0.002
SAR1	-0.949	0.088	-10.827	<0.001
SMA1	-0.707	0.226	-3.131	0.002

Table S2. Estimated parameters of the SARIMA(1,0,1)(1,1,1)$_{12}$ model for the target series from June 2008 to December 2016.

Model	R^2	Log-Likelihood	AIC	BIC	Normalized BIC	Ljung-Box *Q*	
						Statistics	*P*
SARIMA(1,0,1)(1,1,1)$_{12}$	0.864	-1099.009	2208.018	2220.572	21.602	8.761	0.846

Table S3. The goodness of fit test of the preferred SARIMA model for the target series from June 2008 to December 2016.

Lags	Residuals of SARIMA model		Residuals of NAR model		Residuals of LSTM model	
	**Box-Ljung *Q***	***P***	**Box-Ljung *Q***	***P***	**Box-Ljung *Q***	***P***
1	0.098	0.754	0.727	0.394	2.468	0.116
3	0.112	0.990	1.448	0.694	3.467	0.325
6	1.842	0.934	3.647	0.724	6.959	0.325
9	2.761	0.973	4.769	0.854	10.473	0.314
12	4.448	0.974	11.757	0.465	11.179	0.514
15	7.537	0.941	13.361	0.574	16.304	0.362
18	8.761	0.965	13.750	0.745	21.606	0.250
21	9.719	0.982	17.062	0.707	27.494	0.155
24	15.157	0.916	17.108	0.844	29.205	0.213
27	20.138	0.825	17.750	0.911	29.529	0.336
30	22.854	0.821	18.064	0.957	35.149	0.237
33	25.308	0.829	19.470	0.970	41.780	0.141
36	37.095	0.418	20.658	0.981	43.404	0.185

Table S4. Ljung-Box *Q* test of the residuals for the selected three optimal models fitted to the notified HFMD cases series from June 2008 to December 2016 at different lags.

Lags	Observed values		Residuals of SARIMA model		Residuals of NAR model		Residuals of LSTM model	
	LM-test	*P*	LM-test	*P*	LM-test	*P*	LM-test	*P*
1	51.644	<0.001	0.136	0.712	0.086	0.769	0.490	0.484
3	69.573	<0.001	1.369	0.713	1.257	0.739	2.846	0.416
6	68.356	<0.001	3.151	0.790	2.346	0.885	8.744	0.189
9	66.675	<0.001	9.611	0.383	8.095	0.525	8.534	0.481
12	68.750	<0.001	23.007	0.028	9.550	0.655	9.393	0.669
15	66.862	<0.001	22.851	0.087	20.477	0.154	10.494	0.788
18	64.808	<0.001	22.901	0.194	20.832	0.288	17.173	0.511
21	62.946	<0.001	23.073	0.340	21.474	0.430	22.333	0.381
24	69.134	<0.001	16.840	0.855	23.266	0.504	23.755	0.476
27	66.728	<0.001	18.047	0.902	25.234	0.561	27.193	0.453
30	64.059	<0.001	20.507	0.903	30.052	0.463	29.736	0.479
33	61.471	0.002	20.835	0.951	33.224	0.456	36.300	0.317
36	59.046	0.010	31.915	0.663	32.765	0.623	38.356	0.363

Table S5. ARCH effect of the observations and residuals of the selected three models fitted to the notified HFMD cases series from June 2008 to December 2016 with LM test at various lags.

**Parameters**	**Coefficient**	**Standard error**	***t***	***P***
AR1	0.377	0.127	2.968	0.004
MA1	-0.504	0.124	-4.070	<0.001
SAR1	-0.938	0.076	-12.299	<0.001
SMA1	-0.653	0.189	-3.446	0.001

Table S6. Estimated parameters of the SARIMA(1,0,1)(1,1,1)$_{12}$ model for the target series from June 2008 to December 2017.

Model	R^2	Log-Likelihood	AIC	BIC	Normalized BIC	Ljung-Box *Q*	
						Statistics	*P*
SARIMA(1,0,1)(1,1,1)$_{12}$	0.850	-1246.389	2502.777	2515.951	221.621	8.005	0.889

Table S7. The goodness of fit test of the preferred SARIMA model for target series from June 2008 to December 2017.

**Lags**	**Residuals of SARIMA model**		**Residuals of NAR model**		**Residuals of LSTM model**	
	**Box-Ljung *Q***	***P***	**Box-Ljung *Q***	***P***	**Box-Ljung *Q***	***P***
1	0.118	0.732	0.759	0.384	0.224	0.636
3	0.324	0.955	3.863	0.277	5.272	0.153
6	2.104	0.910	5.326	0.503	7.925	0.244
9	2.499	0.981	7.134	0.623	9.273	0.412
12	3.561	0.990	9.655	0.646	9.764	0.637
15	6.688	0.966	10.026	0.818	16.989	0.320
18	8.005	0.979	10.203	0.925	18.592	0.417
21	8.175	0.994	13.032	0.908	19.564	0.549
24	13.966	0.947	13.537	0.956	22.010	0.579
27	21.492	0.763	19.289	0.859	26.655	0.483
30	26.035	0.673	21.828	0.860	29.237	0.505
33	29.036	0.665	22.238	0.922	29.997	0.617
36	36.294	0.455	24.456	0.928	31.024	0.704

Table S8. Ljung-Box *Q* test of the residuals for the selected three optimal models fitted to the notified HFMD cases series from June 2008 to December 2017 at different lags.

Lags	Observed values		Residuals of SARIMA model		Residuals of NAR model		Residuals of LSTM model	
	LM-test	*P*	LM-test	*P*	LM-test	*P*	LM-test	*P*
1	56.835	<0.001	0.149	0.700	0.007	0.935	3.915	0.048
3	76.751	<0.001	1.915	0.590	0.076	0.995	6.659	0.084
6	75.713	<0.001	3.286	0.772	2.735	0.841	10.869	0.093
9	74.335	<0.001	9.033	0.434	3.608	0.935	13.567	0.139
12	76.560	<0.001	21.081	0.049	14.431	0.274	14.193	0.289
15	74.759	<0.001	22.480	0.096	17.071	0.315	17.653	0.281
18	73.311	<0.001	23.535	0.171	20.457	0.308	18.243	0.440
21	71.764	<0.001	24.852	0.254	23.826	0.302	18.519	0.616
24	78.650	<0.001	20.394	0.674	25.552	0.376	21.187	0.628
27	76.543	<0.001	22.363	0.719	26.888	0.470	21.474	0.764
30	73.909	<0.001	25.407	0.705	32.506	0.344	23.564	0.791
33	71.239	<0.001	25.942	0.804	31.970	0.518	26.177	0.795
36	69.310	0.001	33.405	0.593	33.669	0.580	32.374	0.642

Table S9. ARCH effect of the observations and residuals of the selected three models fitted to the notified HFMD cases series from June 2008 to December 2017 with LM test at various lags.

Target series	Hidden units	Delays	MSE*				R			
			Training	Validation	Testing	Overall	Training	Validation	Testing	Overall
TS1	17	5	0.0011	0.0088	0.0068	0.0032	0.990	0.921	0.940	0.971
TS2	19	6	0.0022	0.0035	0.0164	0.0038	0.975	0.964	0.901	0.961

Note: * represents the values of the mean square error(MAE), which are computed based on the processed data with normalized approach; TS1 stands for the reported HFMD cases series from June 2008 to December 2016; TS2 refers to the reported HFMD cases series from June 2008 to December 2017.

Table S10. The preferred NAR models' parameters of various target series.

Target series	Hidden neurons	time steps	MSE*		R		
			Training	Validation	Validation	Testing	Overall
TS1	6	12	0.0022	0.0024	0.982	0.967	0.977
TS2	5	5	0.0031	0.0058	0.974	0.961	0.969

Note: * represents the values of the mean square error(MAE), which are computed based on the processed data with normalized approach; TS1 stands for the reported HFMD cases series from June 2008 to December 2016; TS2 refers to the reported HFMD cases series from June 2008 to December 2017;

Table S11. The preferred LSTM models' parameters of various target series.

Table S12. Future one hundred possible sample paths for the HFMD notified data in mainland China. This Table was provided in a Microsoft Excel.xlsx version on account of the plethora of data.
